# Evaluation of the Diagnostic Potential of Candidate Hypermethylated Genes in Epithelial Ovarian Cancer in North Indian Population

**DOI:** 10.3389/fmolb.2021.719056

**Published:** 2021-10-28

**Authors:** Alka Singh, Sameer Gupta, Manisha Sachan

**Affiliations:** ^1^ Department of Biotechnology, Motilal Nehru National Institute of Technology, Allahabad, India; ^2^ Department of Surgical Oncology, King George Medical University, Lucknow, India

**Keywords:** cell free DNA, early diagnosis, DNA methylation, epigenetic biomarker, epithelial ovarian cancer, liquid biopsy

## Abstract

Most ovarian cancers, despite improvement in management of cancer, are still diagnosed at an advanced stage. Early detection plays an essential role in reducing ovarian cancer mortality and, therefore, is critically needed. Liquid biopsies-based approaches hold significant promise for cancer detection. The present study investigates a panel of epigenetic biomarkers for the detection of epithelial ovarian cancer. A qPCR assay has been developed based on the assessment of DNA methylation markers in circulating cell-free DNA as a minimally invasive tool. Herein, the promoter methylation of seven ovarian cancer-specific genes (RASSF1A, DAPK1, SOX1, HOXA9, HIC1, SPARC, and SFRP1) was analyzed quantitatively in 120 tissue samples by MethyLight assay. The best-performing genes were further evaluated for their methylation status in 70 matched serum cell-free DNA of cancerous and non-cancerous samples. Additionally, DNA methylation patterns of these best-performing genes were validated by clonal bisulfite sequencing. The ROC (Receiver-operator characteristic) curves were constructed to evaluate the diagnostic performances of both individual and combined gene panels. The seven candidate genes displayed a methylation frequency of 61.0–88.0% in tissue samples. The promoter methylation frequencies for all the seven candidate genes were significantly higher in cancer samples than in normal matched controls. In tissue samples, the multiplex MethyLight assay for HOXA9, HIC1, and SOX1 were the best performing gene panels in terms of sensitivity and specificity. The three best-performing genes exhibited individual frequencies of 53.0–71.0% in serum CFDNA, and the multiplex assay for these genes were identified to discriminate serum from cancer patients and healthy individuals (area under the curve: HOXA9+HIC1 = 0.95, HIC1+SOX1 = 0.93 and HOXA9+SOX1 = 0.85). The results of MethyLight showed high concordance with clonal bisulfite sequencing results. Individual genes and combined panel exhibited better discriminatory efficiencies to identify ovarian cancer at various stages of disease when analyzed in tissue and serum cell-free DNA. We report a qPCR-based non-invasive epigenetic biomarker assay with high sensitivity and specificity for OC screening. Our findings also reveal the potential utility of methylation-based detection of circulating cell-free tumor DNA in the clinical management of ovarian cancer.

## Introduction

Ovarian cancer (OC) is the fifth most incident and deadly gynecological malignancy affecting women worldwide, mainly under the age of 55–74. It encompasses heterogeneity at the molecular, clinical, and histopathological level and exhibits the worst prognosis and highest mortality figures for cancer death in women. Approximately 313,959 new cases of ovarian cancer and over 207,252 deaths were estimated to occur worldwide in 2020 (Torre et al., 2018; Sung et al., 2021). While in India, the incidence rate for OC is 5.7 per 100,000 women, affecting 5.4% of all female cancer patients. Overall, this lethal disease accounts for 2.5% of all female associated malignancies but 5% of cancer death in women due to lack of signs and symptoms at the early stage of the disease, lower survival rates mainly driven by advanced stage diagnosis, high recurrence rate, and limited success with existing early diagnosis methods (Siegel et al., 2015).

Epithelial cancers are the most predominant malignant neoplasm accounting for 90% of all OC cases. High-grade serous ovarian carcinoma, characterized by rapid growth with no identified precursor lesions, is the most aggressive tumor and constitutes the most predominant subtype of ovarian cancer, accounting for more than 80% of ovarian cancer-related fatality. Furthermore, no significant improvement to the 5-years survival figures has been observed over the last decade which still hovers at ∼ 35% for advanced stage EOC patients (FIGO stage III/IV). However, diagnosis of disease at preclinical stage (FIGO stage I/II) is highly curable and might substantially improve the survival rates up to 93% (Bowtell et al., 2015; Menon, 2007; Siegel et al., 2011). Early detection is certainly essential to improve the survival rate and effective treatment for better patient outcomes. Therefore it is crucial to identify the most effective and reliable molecular biomarkers involved in ovarian cancer tumorigenesis at the genetic and epigenetic level, which could further aid in the early detection of OC.

Although cancer antigen-125 (CA125) is the most utilized serum-based biochemical marker; its utility as an EOC screening marker is significantly impeded because of high false-positive rate, the lower sensitivity of detection for early stages of EOC, poor specificity as its elevation may also be associated with various benign conditions and several malignancies including lung and colon cancer (Jacobs and Bast, 1989; Woolas et al., 1993). However, the clinical significance of CA125 majorly falls in evaluating and predicting OC recurrence (Rosenthal et al., 2006). To date, only two biochemical markers (CA125 and HE4) have attained approval from the FDA as a marker for monitoring the progression of disease and recurrence. However, these biomarkers are not sufficiently suitable for screening of disease. Furthermore, current conventional screening methods for OC detection exhibit high false-positive rate and lower sensitivity and specificity. Therefore, these methods have failed to overcome the non-specific manifestation of the OC and reduce population mortality morbidity.

Aberrant CpG island hypermethylation in the gene promoter is the most extensively studied epigenetic alternation which is closely associated with transcriptional repression and is involved in carcinogenesis. Furthermore, aberrant methylation patterns represent the earliest carcinogenic event during malignant transformation and constitute the most frequent epigenetic event in cancer progression. Importantly, these altered methylation patterns are sufficiently chemically and biologically stable and can be easily quantified with minimal invasiveness in liquid biopsies (serum/plasma, peritoneal fluids, urine, etc.), constituting a valuable tool to interrogate the presence of tumor DNA and to indicate the presence of the disease prevalence at an earlier stage (Board et al., 2008; Brock et al., 2008; Lofton-Day et al., 2008; Vlassov et al., 2010).

Accumulated evidence from previous studies has identified promoter hypermethylation-induced transcriptional repression of several tumor suppressor genes in ovarian cancer, indicating their potential as a clinically significant biomarker for EOC diagnosis (Balch et al., 2010; Gloss and Samimi, 2014; Nguyen et al., 2014; Singh et al., 2019). Despite several studies on methylation-based diagnostic biomarkers for OC, to date, not even a single epigenetic marker qualifies to preciously and accurately detect the disease in either tumor tissue or minimally invasive body fluid. Additionally, quite a few prior literature indicates the assessment of concurrent methylation of genes promoter in panel towards constructing an assay for EOC diagnosis (de Caceres et al., 2004; Melnikov et al., 2009; Su et al., 2009; Liggett et al., 2011; Zhang et al., 2013). Q-PCR-based multiplex MethyLight assay has been used to quantitate methylation of epigenetic markers in different cancers (Friedrich, 2004; Kang et al., 2008; He et al., 2010; Begum et al., 2011) but has not been reported to be used for developing a biomarker-based assay for early-stage detection of OC. Moreover, detection of differential methylation signatures in serum/plasma with high sensitivity is critically needed for an early EOC diagnosis. Analysis of a panel of potential epigenetic-based biomarkers in liquid biopsies facilitating non-invasive detection of ovarian cancer may add value to the clinical implementation as a sensitive test aiding significantly in clinical decision making (Singh et al., 2019).

Seven tumor suppressor genes (RASSF1A, DAPK1, SOX1, HOXA9, HIC1, SPARC, and SFRP1) were chosen after the extensive screening to construct the multiplex MethyLight assay. We have recently reported HOXA9 and HIC1 as potential markers for non-invasive detection of EOC (Singh et al., 2020). Accumulated evidence from various studies has highlighted extensive frequent hypermethylation-induced downregulation of selected genes under the present study in EOC (Yoon et al., 2001; de Caceres et al., 2004; Takada et al., 2004; Choi et al., 2006; Collins et al., 2006; Melnikov et al., 2009; Socha et al., 2009; Su et al., 2009).

The present study reports the exploration of the performance (the sensitivity and specificity) of epigenetic-based biomarkers, which have been identified to be frequently silenced due to promoter hypermethylation and could potentially serve as a biomarker for non-invasive detection of OC. Herein, we also assessed the feasibility of analyzing hypermethylation trails in cell-free DNA to develop a non-invasive methylation-based assay using a multiplex qPCR assay for early EOC detection. Additionally, site-specific methylation patterns of the best performing genes in tissue samples were validated by clonal bisulfite sequencing to map the consistently hypermethylated CpGs at the promoter region. Subsequently, Receiver operator characteristic (ROC) curves were constructed to assess the diagnostic performances of the selected genes and genes panels to evaluate their clinical utility in predicting EOC at an early stage.

## Material and Methods

### Screening Candidate Genes

A large number of hypermethylated targets have been identified in EOC with reported methylation frequencies which varied widely across several independent studies. Seven tumor suppressor genes (RASSF1A, DAPK1, SOX1, HOXA9, HIC1, SPARC, and SPRF1) were chosen as DNA methylation markers, to construct the multiplex MethyLight assay towards noninvasive detection of EOC at the preclinical stage. The selected candidate genes have been previously reported to be aberrantly hypermethylated in epithelial ovarian cancer particularly serous subtype through gene specific methylation assay as well as genome-wide methylation mapping. Therefore, these genes may qualify as potential candidates to be used in diagnostic biomarker panel.

### Patients and Clinical Samples

Clinical samples comprising of fresh/Formalin-fixed, paraffin-embedded (FFPE) tumor samples and matched peripheral blood specimens were collected randomly upon availability from 120 subjects who underwent surgical resection at the Department of Surgical Oncology, King’s George Medical University, Lucknow, and Motilal Nehru Medical College, Prayagraj since July 2014 till Dec 2019. The sample set included histologically verified primary tumor, and blood samples from ovarian cancer patients (*n* = 85; five benign, six mucinous, and 74 serous EOC) with no prior history of chemotherapy and radiation ranging from FIGO stage I to IV (early stage: I/II (*n* = 20); advanced stage: III/IV (*n* = 65)), and pathologically confirmed healthy control samples (*n* = 35). The median patient age at diagnosis was 50 years (range, 18–70 years). The patient’s clinicopathological characteristics data such as CA125 level, age, menopausal status, tumor size, FIGO stage and histology were retrieved from pathological reports and the patient’s record and are listed in Supplementary File 1: Table S3A,B). The present study was approved by the Institutional ethical committee of Motilal Nehru National Institute of Technology Allahabad (Ethics Committee reference number: IEC/16-17/025), and informed consent was obtained for all the participating patients and control subjects whose samples were analyzed in this study.

All the primary tissue samples were freshly obtained, snap frozen on liquid nitrogen, and further stored at −80°C. 5 ml peripheral blood was collected from participating subjects into BD Vacutainer^®^ separating gel procogulating tubes. Serum was separated by double centrifugation (3500 g for 1 min at 4°C). Serum aliquots were immediately frozen at −80°C.

### DNA Extraction

Fresh-frozen tissue samples (around 10–30 mg) were processed to extract genomic DNA using the standard phenol/chloroform procedure as described previously (Singh et al., 2020). Genomic DNA extraction from FFPE tumor samples was performed with QIAamp^®^ DNA FFPE Tissue kit (Qiagen, Germany) following the protocol described in the user manual.

Cell-free DNA was extracted from 1 ml of serum using MagMax™ Cell-Free DNA isolation kit (Thermo Fisher Scientific, United States) as described previously (Singh et al., 2020), and finally dissolved in 20 µL of LoTE (Life Technologies™, United States). Further, quantification was performed using Qubit^®^ 2.0 Fluorometer (Invitrogen, Life Technologies™, United States) using Qubit^®^ dsDNA HS (High Sensitivity) Assay Kits (Invitrogen, Life Technologies™, United States) and Agilent 2,100 Bioanalyzer (Agilent Technologies, Germany) using Agilent High Sensitivity DNA Kit (Agilent Technologies, Germany). Around 50 ng serum CFDNA was extracted per ml of serum.

### Bisulfite Modification

2 µg of tissue DNA and entire extracted serum CFDNA was used as input material for bisulfite treatment which was performed using the Premium Bisulfite kit (Diagenode, Belgium) as described previously (Singh et al., 2020). Following the bisulfite conversion, all DNA samples were stored at −20°C.

### MethyLight Assay

RASSF1A, DAPK1, SOX1, SPARC, and SFRP1 promoter methylation were analyzed by MethyLight assay in tissue and serum CFDNA samples. HOXA9 and HIC1 have previously been reported (Singh et al., 2020). Two sets of primers and probes designed particularly for bisulfite-treated DNA were used: a methylated set for the candidate genes under study and a reference set for the COL2A1 gene to normalize for the input DNA; primer and probe sequences were purchased from Eurogentec, Belgium. The sequences are listed in (Supplementary File 1: Table S1A). The primer and probe for COL2A1 were designed as previously published studies (Friedrich, 2004; Ogino et al., 2006; Kang et al., 2008; Shih et al., 2013; Liu et al., 2014).

MethyLight PCR was carried out in duplicates in a reaction volume of 10 µL including 0.5 µM each of forward and reverse PCR primers; 0.2 µM probe; 1 µL of bisulfite-modified DNA, and 1x Takyon Rox Probe MasterMix dTTP Blue (Eurogentec, Belgium). Fragments were amplified at 95°C for 4 min, then 50 cycles of 95°C for 15 s followed by annealing/extension at 60°C for 35 s using the ABI StepOne Plus system (Thermo Fisher Scientific, United States). The median value was used for data analysis. Multiplex reactions were carried out with the same primers and probes and under similar amplification conditions as singleplex MethyLight assay.

Bisulfite converted fully methylated M. SssI-treated human genomic DNA served as a positive reference for the qMSP reaction, and standard curves were generated from serial dilutions (1:5 series) of the positive control for quantification, as described previously (Singh et al., 2020). The qMSP output was determined as percent of methylated reference (PMR) values in accordance with previously published reports (Eads et al., 2000; He et al., 2010; Reinert et al., 2012; Andresen et al., 2015; Olkhov-Mitsel et al., 2015). Briefly, the median Gene: COL2A1 ratio of the sample was divided by the median Gene: COL2A1 ratio of the fully methylated positive control and multiplied by 100.

For DNA methylation analysis, no standard cutoff value has so far been defined to categorize the methylated and unmethylated loci. In order to ensure high specificity, the highest PMR value across all normal control samples and all genes was used to fix the threshold (cutoff value = 4) for scoring positive methylation, irrespective of the gene in question as described previously (Singh et al., 2020). The samples with PMR values below the scoring thresholds were designated as “unmethylated” (methylation negative) and those with PMR values higher than the scoring threshold were designated as “Methylated” (methylation positive).

### Clonal Bisulfite Sequencing

The methylation status of CGs in the promoter region of the best performing genes (HOXA9, SOX1, and HIC1) in representative tissue samples (OC, *n* = 5 (serous histology) and normal, *n* = 3) was validated by clonal bisulfite sequencing. The bisulfite primers targeting the respective gene promoter region, in particular flanking the section assessed by the MethyLight assay were used for PCR as described previously (Singh et al., 2020). The sequences of clonal bisulfite sequencing primers and PCR conditions are listed in (Supplementary File 1: Table S1B). The PCR product was purified with QIAquick gel extraction kit (Qiagen, United States) and cloned into T-vector using an InsTAclone™ PCR cloning kit (Thermo Fisher Scientific, United States), and sequenced. Blue-white screening was performed to pick for positive transformed clones, and for each PCR product, 10-12 independent randomly chosen clones were sequenced using 3730XL Genetic Analyzer (Thermo Fisher Scientific, United States).

### Statistics Analysis

For statistical analysis, GraphPad Prism V5.0 (GraphPad Software, San Diego, CA) and R version 3.4.4 software were used. pheatmap package in R was used to construct the Heatmap. Microsoft Excel 2007 was used for PMR value calculation. Mann-Whitney U test and Student *t*-test were used to evaluate the potential association between promoter hypermethylation and patient’s clinicopathological characteristics features. Pearson’s χ^2^ or Fisher’s extract test were used for comparison of categorical variables. Relative gene expression was calculated by 2^−ΔΔCT^ (Livak method). Percent methylation reference values were used as input to generate Receiver operator characteristic (ROC) curves to assess the accuracy of methylation levels in detecting OC. Logistic regression model was used to construct ROC curves to discriminate cancers from healthy controls. Pairwise comparison of ROC curves to access the accuracy of prediction of marker panels was performed by DeLong’s test. Both sensitivity and specificity were considered significant parameters to define the optimal cutoff value (highest Youden Index) and accuracy. Clonal bisulfite sequencing results were analyzed in Chromas (Technelysium Pty Ltd., Australia). *p*-values of <0.05 were defined as statistically significant.

## Results

### MethyLight Assay Development and Standardization in Tissue

The promoter methylation levels of selected candidate genes (RASSF1A, DAPK1, SOX1, HOXA9, HIC1, SPARC, and SFRP1) were quantitatively evaluated by MethyLight in tumor DNA samples of patients with EOC. To examine the linearity and accuracy of the qMSP assay, standard curves for COL2A1, DAPK1, SOX1, RASSF1A, HOXA9, SFRP1, SPARC, and HIC1 were generated with the fully-methylated positive control (Supplementary File 1: Figure S1). Correlation coefficients (*R* ^2^) for the test of a linear association were 0.9952, 0.9963, 0.9628, 0.9341, 0.9790 and 0.9699 for COL2A1, DAPK1, SOX1, RASSF1A, SFRP1, and SPARC respectively. The standard curve for HOXA9 and HIC1 gene (*R* ^2^ = 0.9672 and 0.9657) has been previously reported (Singh et al., 2020). These findings indicate good reproducibility and high sensitivity of detection in clinical samples. A representation of methylation detection in tumor samples by qMSP for all the candidate genes in singleplex and multiplex assays is presented in (Supplementary File 1: Figure S2A).

A significantly strong correlation between the normalized ratio of the singleplex and multiplex assay was observed for all the selected genes under investigation in the present study, signifying equivalent performance in facilitating methylation detection in these clinical samples (Supplementary File 1: Figure S3).

### Methylation Status of Candidate Genes in Primary Tumor

**FIGURE 1:**
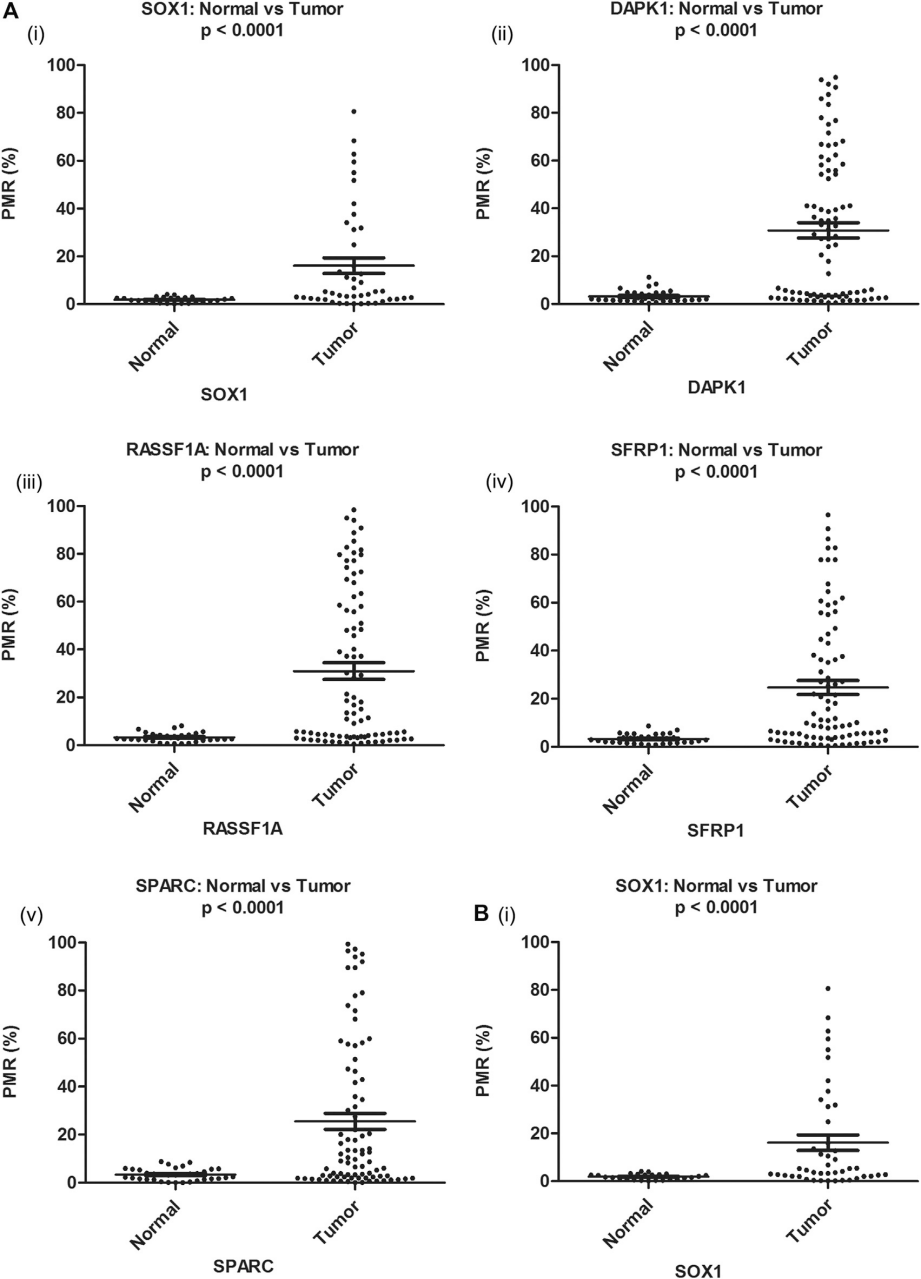
Distribution of PMR value of candidate genes in EOC and corresponding noncancerous normal tissue samples **(Ai–v)** and serum samples **(Bi)** respectively. Distribution of PMR value of HOXA9 and HIC1 gene in EOC and corresponding noncancerous normal tissue and serum samples has been published (Singh et al., 2020). PMR: percentage of methylated reference.

To evaluate cancer-specificity, the promoter methylation levels of RASSF1A, DAPK1, SOX1, HOXA9, HIC1, SPARC, and SFRP1 were quantitatively analyzed by MethyLight in singleplex and multiplex assays. The distribution of PMR values for each gene under investigation is presented in (Figure 1).

The median promoter methylation level in tumor DNA of OC patients and normal control subjects was 19.64 (95% CI, 5.17-60.53) and 2.14 (95% CI, 1.46-3.81) respectively for SOX1; 15.10 (95% CI, 3.60-58.28) and 2.97 (95% CI, 2.10-4.20) respectively for RASSF1A; 27.19 (95% CI, 3.13-55.90) and 2.24 (95% CI, 1.48-4.67) respectively for DAPK1; 10.09 (95% CI, 3.93-40.62) and 3.17 (95% CI, 1.78-4.19) respectively for SFRP1 and 10.43 (95% CI, 2.36-44.66) and 3.12 (95% CI, 1.40-5.59) respectively for SPARC (Table 1). The methylation level of HOXA9 and HIC1 gene in tumor samples of patients with epithelial ovarian carcinoma and healthy control group has been previously described (Singh et al., 2020)**.**

**TABLE 1:** PMR value of candidate genes in tissue and serum samples of patients with epithelial ovarian carcinoma and healthy control group. PMR: percentage of methylated reference. *p*-value: Mann-Whitney U two-tailed tests. ^a^The PMR value of HOXA9 and HIC1 gene in tissue and serum samples of patients with epithelial ovarian carcinoma and healthy control group has been previously described (Singh et al., 2020).

Gene	Parameter	No. of patients	Mean ± Std.Error	Min	Max	25th	50th	75th	*p*-value
**HOXA9^a^**	**Tissue PMR**								
	Tumor	85	32.83 ± 3.02	0.29	97.54	8.78	24.72	49.34	<0.0001***
	Healthy	35	2.72 ± 0.31	0.14	6.68	1.32	2.53	3.8	
	**Serum PMR**								
	Tumor	45	13.2 ± 2.52	0.05	87.42	1.97	7.45	18.95	<0.0001***
	Healthy	25	1.45 ± 0.21	0.18	3.57	0.46	1.35	2.30	
**HIC1^a^**	**Tissue PMR**								
	Tumor	85	28.54 ± 2.72	0.14	88.82	8.07	22.05	45.98	<0.0001***
	Healthy	35	3.47 ± 0.39	0.13	10.65	1.97	3.33	3.93	
	**Serum PMR**								
	Tumor	45	12.98 ± 2.21	0.06	58.68	3.28	7.96	14.91	<0.0001***
	Healthy	25	1.67 ± 0.26	0.06	3.79	0.35	1.58	2.78	
**SOX1**	**Tissue PMR**								
	Tumor	85	33.24 ± 3.35	0.45	96.53	5.17	19.64	60.53	<0.0001***
	Healthy	35	2.76 ± 0.29	0.74	6.52	1.46	2.14	3.81	
	**Serum PMR**								
	Tumor	45	16.09 ± 3.25	0.19	80.61	2.14	4.60	28.04	<0.0001***
	Healthy	25	1.84 ± 0.24	0.22	4.15	0.61	1.73	2.72	
**RASSF1A**	**Tissue PMR**								
	Tumor	85	30.97 ± 3.46	0.49	98.42	3.60	15.10	58.28	<0.0001***
	Healthy	35	3.24 ± 0.31	0.59	8.09	2.10	2.97	4.20	
**DAPK1**	**Tissue PMR**								
	Tumor	85	30.82 ± 3.24	0.51	94.87	3.13	27.19	55.9	<0.0001***
	Healthy	35	3.11 ± 0.41	0.33	11.18	1.48	2.24	4.67	
**SFRP1**	**Tissue PMR**								
	Tumor	85	24.71 ± 2.94	0.34	96.53	3.93	10.09	40.62	<0.0001***
	Healthy	35	3.30 ± 0.32	0.87	8.72	1.78	3.17	4.19	
**SPARC**	**Tissue PMR**								
	Tumor	85	25.55 ± 3.36	0.03	99.38	2.36	10.43	44.66	<0.0001***
	Healthy	35	3.39 ± 0.42	0.05	8.77	1.40	3.12	5.59	

DNA methylation of HOXA9, HIC1, DAPK1, SOX1, RASSF1A, SFRP1 and SPARC was observed in 82.3% (70/85), 80.0% (68/85), 69.4% (59/85), 78.8% (67/85), 71.8% (61/85), 74.1% (63/85) and 61.2% (52/85) of the tissue samples of patients with EOC. Nevertheless, these seven markers were found to be methylated in only 14.3% (5/35), 22.9% (8/35), 28.6% (10/35), 20.0% (7/35), 28.6% (10/35), 28.6% (10/35) and 31.4% (11/35) of healthy control samples.

**FIGURE 2:**
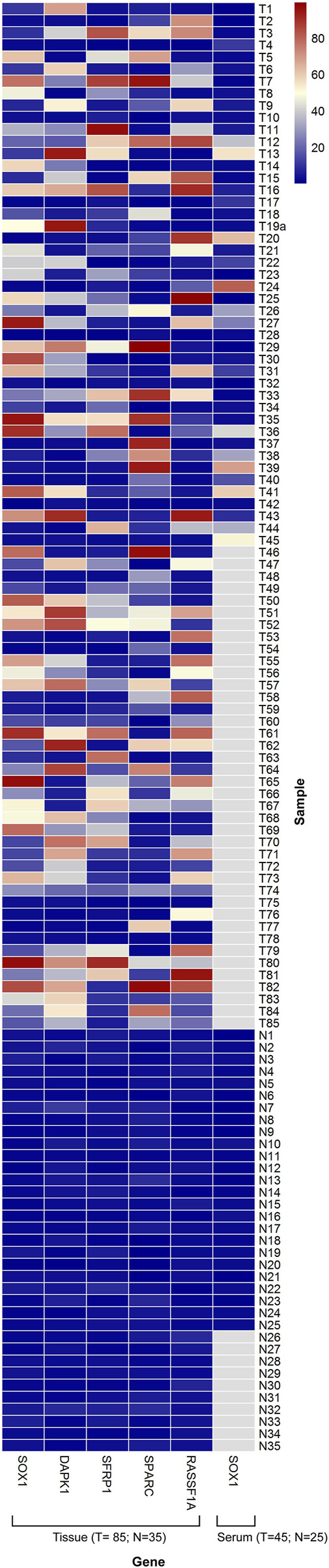
Heat map showing distribution of PMR values for the candidate biomarker genes SOX1, DAPK1, SFRP1, SPARC and RASSF1A. Representations of Methylation level of candidate genes in tissue DNA (85 EOC/35 Control) and in serum cell free DNA (45 EOC/25 Control). The range of PMR levels which corresponds to different colors in the figure is shown. White squares (N/A) indicate that data were not available for those samples. PMR value data for HOXA9 and HIC1 in tissue and serum samples has been published elsewhere (Singh et al., 2020).

The methylation level in the methylated EOC tumor samples ranged from 4 to 97% for HOXA9; 7–89% for HIC1; 4–97% for SOX1; 4–95% for DAPK1; 5–97% for SFRP1; 6–99% for SPARC and 4–98% for RASSF1A gene, respectively. However, most of the unmethylated healthy samples reflected methylation levels in the range of 0–3% (Figure 2).

For all the seven candidate genes, a significantly higher promoter methylation frequency was observed in tumor samples of patients with OC in comparison to the normal control samples (Pearson chi-square: HOXA9, *p* < 0.00001; HIC1, *p* < 0.00001; DAPK1, *p* = 0.00004; SOX1, *p*=<0.00001; RASSF1A, *p* = 0.00001; SFRP1, *p* < 0.00001 and SPARC, *p* = 0.00302 respectively) (Table 2).

**TABLE 2:** Performance assessment of promoter methylation as biomarker for detection of EOC in tissue and serum samples. Table illustrates the Sensitivity, Specificity, AUC, Accuracy, Optimal Cutoff and Methylation Index of singleplex and multiplex MethyLight assays for the detection of control and epithelial ovarian cancer patients. AUC: Area under the curve; PPV: Positive predictive value; NPV: Negative prediction value; MI: Methylation Index; *p*-value: Mann-Whitney U test.

Marker	Sensitivity	Specificity	AUC	Optimal cutoff	PPV	NPV	Accuracy	*p*-value	Methylation index (MI)
**TISSUE**									
HOXA9**^a^**	82.35	85.71	0.92	0.77	99.33	66.67	86.6	<0.00001	0.82
HIC1**^a^**	80.00	77.14	0.85	0.68	89.5	61.4	82.5	<0.00001	0.80
DAPK1	69.41	71.43	0.81	0.49	85.51	49.02	75.8	0.00004	0.69
SOX1	78.82	80.00	0.88	0.38	90.54	60.87	80.8	<0.00001	0.78
RASSF1A	71.76	71.43	0.78	0.42	85.92	51.02	73.3	0.00001	0.72
SFRP1	74.12	71.43	0.80	0.66	86.30	53.19	75.0	<0.00001	0.74
SPARC	61.18	68.57	0.73	0.47	82.54	42.11	73.3	0.00302	0.61
HOXA9+HIC1**^a^**	88.24	88.57	0.92	0.49	94.9	75.6	88.3	<0.00001	0.88
HOXA9+SOX1	85.88	88.57	0.92	0.48	94.81	72.09	87.0	<0.00001	0.86
SOX1+HIC1	83.53	88.57	0.89	0.72	94.67	68.89	87.5	<0.00001	0.83
HIC1+SFRP1	78.82	77.14	0.87	0.75	89.33	60.00	84.0	<0.00001	0.79
RASSF1A+HOXA9	76.47	80.00	0.87	0.49	90.28	58.33	87.5	<0.00001	0.76
DAPK1+SOX1	72.94	77.14	0.87	0.45	88.57	54.00	80.0	<0.00001	0.73
**SERUM**									
HOXA9^**a**^	62.22	100	0.81	0.70	100	59.52	77.1	<0.00001	0.62
HIC1^**a**^	71.11	100	0.88	0.71	100	65.75	82.8	<0.00001	0.71
SOX1	53.33	96	0.77	0.62	96.0	53.33	72.8	0.00003	0.53
HOXA9+SOX1	66.67	96	0.85	0.70	96.77	61.54	88.0	<0.00001	0.67
HOXA9+HIC1**^a^**	88.89	100	0.95	0.64	100	83.33	92.8	<0.00001	0.89
SOX1+HIC1	80.00	96	0.93	0.54	97.30	72.73	87.0	<0.00001	0.80

a Diagnostic significance of DNA methylation markers-HOXA9 and HIC1 in singleplex and multiplex MethyLight assay analyzed in tissue and serum samples has been previously published (Singh et al., 2020).

### Identification of Best-Performing Gene/Gene Panel in Tissue Samples

In the singleplex assay, high methylation (>70% of EOC samples) was evident for HOXA9, HIC1, SOX1, SFRP1, and RASSF1A (in decreasing order of methylation frequency). DAPK1 and SPARC revealed the presence of methylation with a sensitivity of 69.41 and 61.18% and a specificity of 71.43 and 68.57%, respectively (AUC = 0.81 and 0.73, respectively). The highest sensitivity and specificity was shown by HOXA9 (82.35 and 85.71%, respectively; AUC = 0.92), while SPARC presented the lowest sensitivity and specificity. The methylation status of biomarkers: HIC1, SOX1, SFRP1 and RASSF1A in a series of EOC tumors versus non-malignant control samples resulted in a sensitivity of 80.0, 78.82, 74.12 and 71.76%, respectively and a specificity of 77.14, 80.0, 71.43 and 71.43%, respectively (AUC = 0.85, 0.88, 0.80 and 0.78, respectively) (Table 2).

The cutoff value of methylation level of the seven candidate gene (RASSF1A, DAPK1, SOX1, HOXA9, HIC1, SPARC, and SFRP1), calculated using Youden index formula was 0.42, 0.49, 0.38, 0.77, 0.68, 0.47 and 0.66, respectively. The PPV, NPV and accuracy for these genes were, for RASSF1A, 85.92, 51.02 and 73.30%; for DAPK1, 85.51, 49.02 and 75.8%; for SOX1, 90.54, 60.87 and 80.8%; for HOXA9, 99.33, 66.67 and 86.6%; for HIC1, 89.5, 61.4 and 82.5%; for SPARC, 82.54, 42.11 and 73.30%; and for SFRP1, 86.30, 53.19 and 75.0%, respectively (Table 2 and Supplementary File 1: Figure S4). The diagnostic implication of methylation-based epigenetic markers-HOXA9 and HIC1 in singleplex and multiplex MethyLight assay has been previously published (Singh et al., 2020).

The two-gene combination marker panel was evaluated through multiplex MethyLight assay to narrow down the best performing genes for their further validation of diagnostic significance in serum samples of EOC patients. The use of marker panel highlighted the demonstrable differences in the discriminatory power of both the genes compared to the utility of a single gene by itself.

**FIGURE 3:**
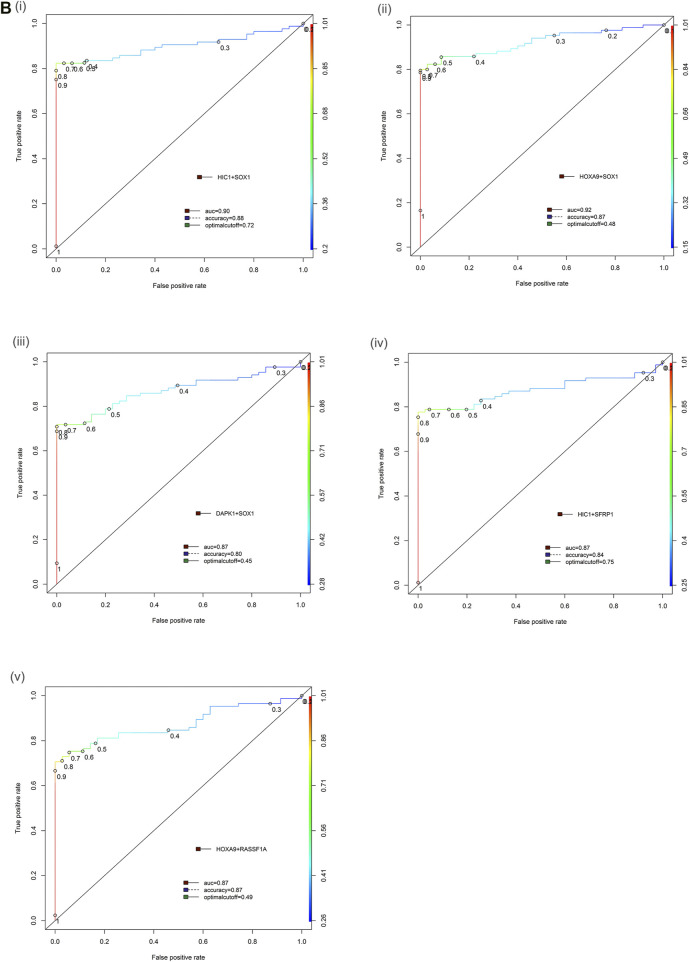
Receiver operator characteristic (ROC) curve differentiating EOC patients from healthy control in tissue samples. The area under the ROC curve (AUC) suggests the accuracy of the biomarkers in distinguishing ovarian carcinoma from normal healthy control sample, is depicted for the biomarkers panels **(Bi)** HIC1+SOX1, **(ii)** HOXA9+SOX1, **(iii)** DAPK1+SOX1, **(iv)** HIC1+SFRP1 and **(v)** HOXA9+RASSF1A, respectively. [Combined two DNA methylation marker panel] which is based on the sum of the two PMR values. AUC: area under the curve. The ROC curve for biomarker panel HOXA9+HIC1 in tissue samples has been previously published (Singh et al., 2020).

In the multiplex assay, when either or both gene promoter exhibited positive methylation, the highly sensitive and specific discrimination of EOC patients from normal controls was observed for HOXA9 + HIC1 (88.24% sensitivity, 88.57% specificity; AUC = 0.92 (previously published (Singh et al., 2020))**,** HOXA9 + SOX1 (85.88% sensitivity, 88.57% specificity; AUC = 0.92) and HIC1 +SOX1 (83.53% sensitivity, 88.57% specificity; AUC = 0.89), respectively. Simultaneous methylation of HIC1 + SFRP1 and DAPK1 + SOX1 exhibited sensitivity of 78.82 and 72.94%, respectively with specificity of 77.14% (AUC = 0.87 for both gene panels) for cancer detection. The combined methylated HOXA9 + RASSF1A achieved 76.47% sensitivity and 80.0% specificity with AUC of 0.87 (Table 2 and Figure 3).

Methylated HOXA9, SOX1, and HIC1 exhibited the highest sensitivity, specificity, AUC, and accuracy for cancer detection in both singleplex and multiplex MethyLight assay, thereby asserting that their promoter hypermethylation was highly cancer-specific. HOXA9, HIC1, and SOX1 were the best performing gene panels in tissue samples and were screened to validate their diagnostic relevance in serum samples from EOC patients**.**

We further cross-validated the performance of the multiplex two-gene marker panel in discriminating cancer vs normal by data-partitioning (training data set and validation data set) randomly using decision tree analysis. The threshold value to discriminate cancer and normal was initially determined in training data set and then the analysis model was further validated in validation data set to calculate the accuracy, misclassification error and Goodness of fit. There was no significant difference between the two data sets with respect to any of the clinicopathological characteristics among the cases. The performance of all two-gene marker panels was found significant and the gene panels differentiated cancer vs normal with an higher accuracy in tissue samples (Supplementary File 1: Table S2). The performance of two-gene marker panel HOXA9 and HIC1 in tissue samples by data partitioning into training data set and test data set randomly using decision tree analysis has been previously published (Singh et al., 2020).

### Methylation Status in Various Stages of EOC

All the genes under study were found highly positive for methylation at early stages (Stage I/II), thereby reflecting their potential to identify the early stage of the disease and serve as an early detection marker. Methylation of HOXA9 was found positive in 80.0% (16/20) patients with stage I/II cancer, 81.5% (44/54) of stage III, and 90.9% (10/11) of stage IV ovarian cancer patients. Methylated HIC1 and SOX1 exhibited a similar trend to identify ovarian cancer at various stages of disease (70.0% (14/20) of stage I/II, 85.2% (46/54) and 83.3% (45/54) of stage III, respectively, and 72.7% (8/11) of stage IV ovarian cancers).

Higher sensitivity in identifying the early stages of ovarian cancer was exhibited by each of the two-gene combination marker panels. The combined panel of HOXA9 + HIC1 showed the best discriminatory efficiencies at all stages of ovarian cancer (80.0% (16/20) of stage I/II, 90.7% (49/54) of stage III, and 90.9% (10/11) of stage IV of ovarian cancer). Simultaneous methylation of SOX1+HOXA9 exhibited positivity for methylation in 75.0% (15/20) of stage I/II, 85.2% (46/54) of stage III, and 81.8% (9/11) of stage IV of ovarian cancer. Methylation frequency of other genes and combined two-gene panels to detect cancer at different stages of epithelial ovarian cancer has been described in (Table 3).

**TABLE 3:** Methylation frequency of candidate gene and two-gene marker panels in different stages of epithelial ovarian carcinoma.

	Gene	Stage I/II (%)		Stage III (%)		Stage IV (%)	
		Positive/Total	Positive (%)	Positive/Total	Positive (%)	Positive/Total	Positive (%)
**TISSUE**	HOXA9	16/20	80	44/54	81.5	10/11	90.9
	HIC1	14/20	70	46/54	85.2	8/11	72.7
	SOX1	14/20	70	45/54	83.3	8/11	72.7
	RASSF1A	14/20	70	38/54	70.4	9/11	81.8
	SFRP1	14/20	70	41/54	75.9	8/11	72.7
	SPARC	11/20	55	35/54	64.8	6/11	54.5
	DAPK1	14/20	70	39/54	72.2	6/11	54.5
	HOXA9+HIC1	16/20	80	49/54	90.7	10/11	90.9
	HIC1+SOX1	14/20	70	47/54	87.0	9/11	81.8
	SOX1+HOXA9	15/20	75	46/54	85.2	9/11	81.8
	HOXA9+RASSF1A	13/20	65	42/54	77.8	9/11	81.8
	HIC1+SFRP1	14/20	70	45/54	83.3	8/11	72.7
	DAPK1+SOX1	14/20	70	41/54	75.9	6/11	54.5
**SERUM**	HOXA9	7/10	70	17/30	56.7	4/5	80
	HIC1	7/10	70	21/30	70	4/5	80
	SOX1	7/10	70	13/30	43.3	4/5	80
	HOXA9+HIC1	10/10	100	24/30	80	4/5	80
	HIC1+SOX1	10/10	100	21/30	70	5/5	100
	SOX1+HOXA9	8/10	80	17/30	56.7	4/5	80

No significant differences in the promoter methylation levels of all candidate genes/marker panels were apparent across the various pathological stage and histological subtypes. Nevertheless, a significant correlation was observed between the promoter hypermethylation of all candidate genes and marker panels and the age of the patient and their menopausal status, as evident from (Table 4).

**TABLE 4:** Association of promoter hypermethylation status of candidate genes with patient’s clinico-pathological features in EOC tissue samples. *p*-value: Pearson’s chi-square and Fisher’s exact tests. Abbreviation: Pos: biomarker panel positive for methylation; Neg: biomarker panel negative for methylation; NS: not significant. A positive biomarker panel is defined as when either or both gene promoter show methylation.

Tissue	No. of patients	HOXA9^a^		HIC1^a^		SOX1		RASSF1A		SFRP1		SPARC		DAPK1	
Feature		M	U	M	U	M	U	M	U	M	U	M	U	M	U
**Tumor**	85	70	15	68	17	67	18	61	24	63	22	52	33	59	26
**Healthy**	35	5	30	8	27	7	28	10	25	10	25	11	24	10	25
**P value**		<0.0001***		<0.0001***		< 0.0001***		< 0.0001***		< 0.0001***		0.0046**		<0.0001***	
**Histology**															
**Serous**	74	61	13	58	16	58	16	53	21	53	21	47	27	51	23
**Mucinous**	6	5	1	6	0	5	1	5	1	6	0	5	1	4	2
**Benign**	5	4	1	4	1	4	1	3	2	4	1	0	5	4	1
**P value**		**NS**		**NS**		**NS**		**NS**		**NS**		0.0096**		**NS**	
**FIGO stage**															
**I/II**	20	16	4	14	6	14	6	14	6	14	6	11	9	14	6
**III/IV**	65	54	11	54	11	53	12	47	18	49	16	41	24	45	20
**P value**		**NS**		**NS**		**NS**		**NS**		**NS**		**NS**		**NS**	
**Age (Median =50)**															
**≥ Median age**	64	31	33	33	31	34	30	33	31	34	30	32	32	34	30
**< Median age**	56	44	12	43	13	41	15	38	18	39	17	31	25	35	21
**P value**		0.0007***		0.0047**		0.0253*		**NS**		**NS**		**NS**		**NS**	
**Menopausal status**															
**Pre**	53	41	12	40	13	40	13	36	17	38	15	29	24	35	18
**Post**	67	34	33	36	31	35	32	35	32	35	32	34	33	34	33
**P value**		0.0042**		0.0216*		0.0132*		**NS**		0.0385*		**NS**		**NS**	

Tissue	No. of patients	HOXA9 + HIC1^a^		HOXA9 + SOX1		HIC1+ SOX1		HOXA9+ RASSF1A		HIC1+ SFRP1		DAPK1+ SOX1	
Feature		Pos	Neg	Pos	Neg	Pos	Neg	Pos	Neg	Pos	Neg	Pos	Neg
**Tumor**	85	75	10	73	12	71	14	65	20	67	18	62	23
**Healthy**	35	4	31	4	31	4	31	7	28	8	27	8	27
**P value**		<0.00001***		< 0.0001***		< 0.0001***		< 0.0001***		< 0.0001***		< 0.0001***	
**Histology**													
**Serous**	74	65	9	61	13	60	14	57	17	57	17	53	21
**Mucinous**	6	6	0	5	1	6	0	5	1	6	0	4	2
**Benign**	5	4	1	4	1	4	1	2	3	4	1	4	1
**P value**		**NS**		**NS**		**NS**		**NS**		**NS**		**NS**	
**FIGO stage**													
**I/II**	20	16	4	15	5	14	6	13	7	14	6	14	6
**III/IV**	65	59	6	55	10	56	9	51	14	53	12	47	18
**P value**		**NS**		**NS**		**NS**		**NS**		**NS**		**NS**	
**Age (Median =50)**													
**≥ Median age**	64	33	31	31	33	31	33	31	33	32	32	30	34
**< Median age**	56	48	8	43	13	43	13	39	17	43	13	37	19
**P value**		<0.0001***		0.0024**		0.0024**		0.0258*		0.0027**		0.0432*	
**Menopausal status**													
**Pre**	53	45	8	40	13	41	12	37	16	40	13	37	16
**Post**	67	36	31	34	33	33	34	33	34	35	32	30	37
**P value**		0.0004***		0.0079**		0.0023**		0.0265*		0.0132*		0.0093**	

a Promoter hypermethylation of DNA methylation markers-HOXA9 and HIC1 in singleplex and multiplex MethyLight assay analyzed in tissue samples has been previously published (Singh et al., 2020).

### Methylation Mapping of the Best-Performing Gene in Tissue Samples by Clonal Bisulfite Sequencing

Methylation pattern was analyzed in ovarian cancer tissue samples through clonal sodium bisulfite DNA sequencing in the promoter region harboring a total of 19, 14, and 26 CpG sites for HOXA9, HIC1, and SOX1 gene, respectively. The clonal bisulfite sequencing results for HOXA9 and HIC1 have been published previously, which strongly confirms the promoter hypermethylation status of these genes as assessed by qMSP in EOC (Singh et al., 2020).

**FIGURE 4:**
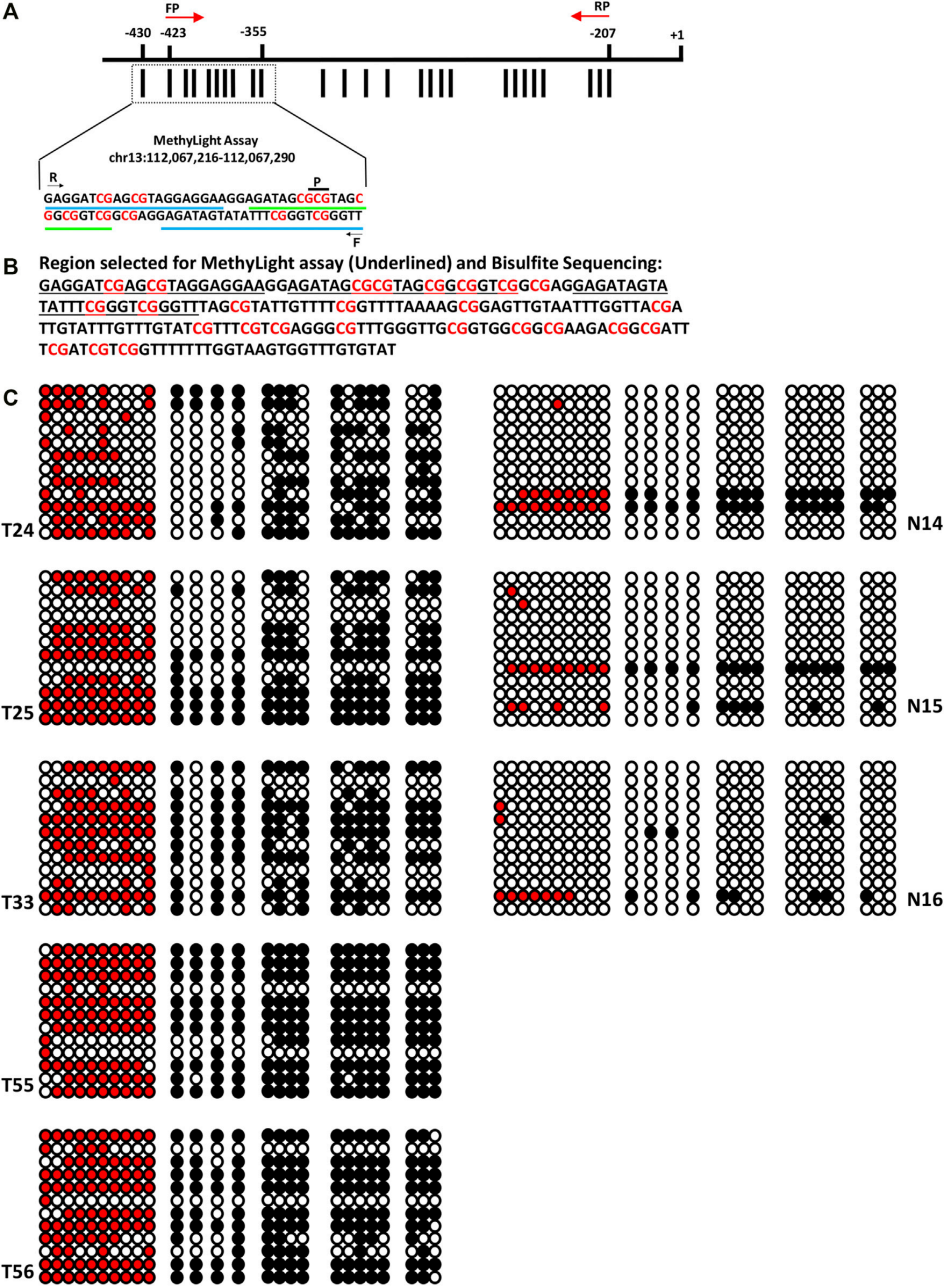
Clonal Bisulfite sequencing of SOX1 in normal and EOC tissues. **(A)** Promoter region of SOX1 comprising 26 CpG sites. The distribution of CpG sites (vertical bar) and position of the hybridized PCR primers (F: forward primer, R: reverse primer, *p*: probe) for MethyLight assay and for clonal Bisulfite sequencing (indicated with red color arrow) are shown. **(B)** Nucleotide sequence of the promoter region of SOX1 for MethyLight and bisulfite sequencing. **(C)** Clonal bisulfite sequencing results for normal and malignant tissues samples of EOC. For each selected tissue sample, 12 randomly chosen clones were sequenced and the methylation status all 26CpG sites is indicated by the circles: closed (black) and open circles represent methylated and unmethylated CpG sites, respectively. Closed (red) represents methylated CpGs in the region analyzed by MethyLight assay.

Bisulfite sequencing confirms the promoter hypermethylation status of SOX1 in EOC, as assessed by MethyLight. Highly dense methylation was observed in the tumor samples, particularly in the region assessed by qMSP. However, no hypermethylation was observed in the normal samples. As expected, malignant samples with higher PMR values reflected substantially dense methylated CpG sites. In contrast, the normal samples with lower PMR values displayed few methylated CpG sites. Additionally, 10-fold higher methylation was observed in tumor tissue compared to their normal counterpart (Figure 4).

### MethyLight Assay Development and Standardization in Serum

**FIGURE 5:**
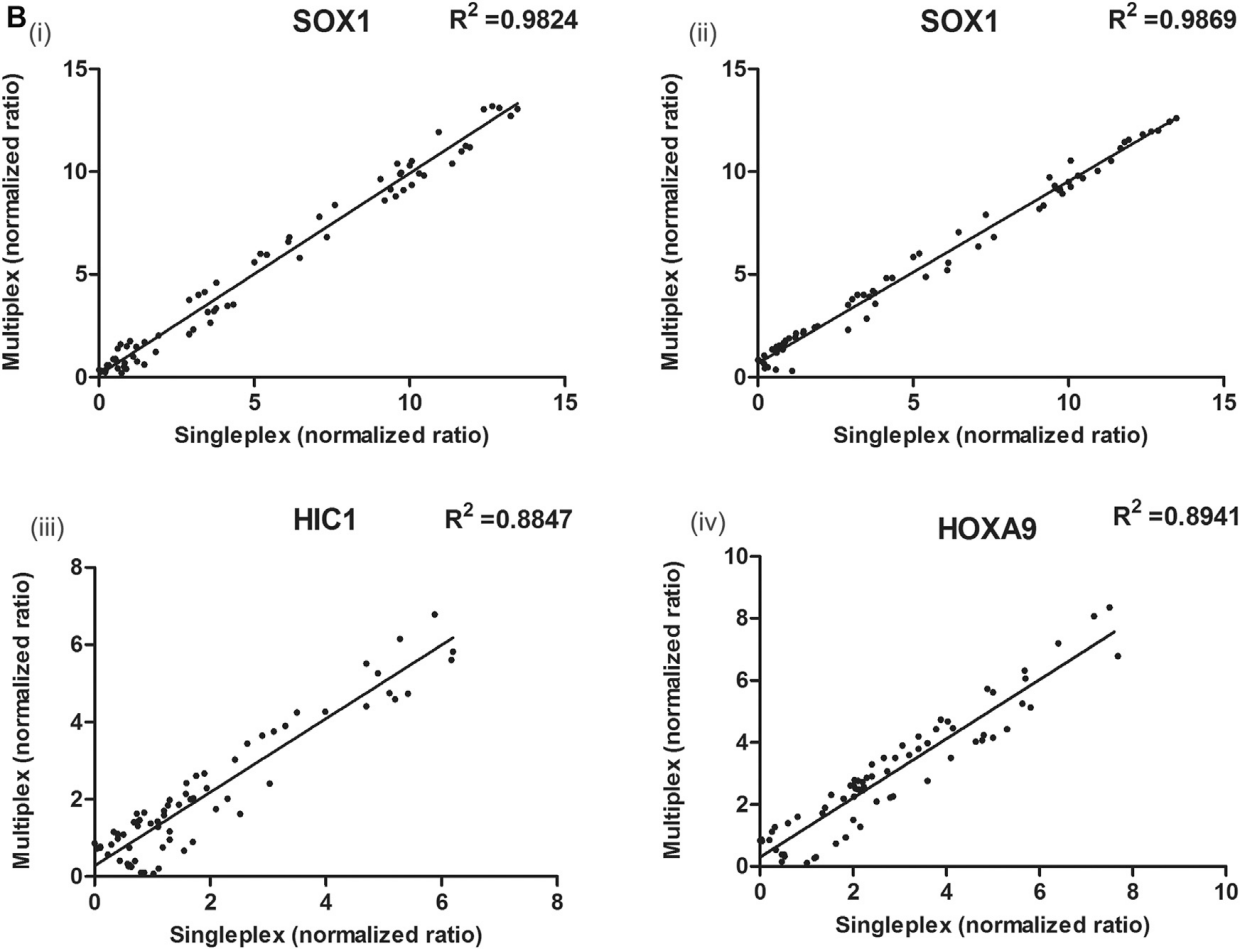
Correlation between the ΔCq values obtained with singleplex and multiplex assays for **(Bi,ii)** SOX1 (multiplexed with HIC1 and HOXA9), **(iii)** HIC1 (multiplexed with SOX1) and **(iv)** HOXA9 (multiplexed with SOX1) genes, respectively in serum samples. The ΔCq values between singleplex and multiplex MethyLight assay differ due to differences in set thresholds. The normalized ratios of singleplex and multiplex MethyLight assay for HOXA9 (multiplexed with HIC1) and HIC1 (multiplexed with HOXA9) in serum samples has been previously published (Singh et al., 2020).

To validate cancer specificity, performance, and utility as an efficient non-invasive biomarker, the promoter methylation levels of best-performing genes (HOXA9, HIC, and SOX1) were evaluated in matched serum CFDNA samples of patients with EOC. A representation of methylation detection by qMSP for these genes in singleplex and multiplex assays is presented in (Supplementary File 1: Figure S2B). A strong correlation between the normalized ratio of the singleplex and multiplex assay was observed for these markers, indicating equivalent performance in facilitating methylation detection (Figure 5).

### Methylation Status of Best Performing Genes in Matched Serum Cell-Free DNA

The distribution of PMR values for SOX1 is demonstrated in (Figure 1). The median promoter methylation level of SOX1 in CFDNA of OC patients was 4.60 (95% CI, 2.13-28.04), whereas that of healthy control was 1.73 (95% CI, 0.61-2.72), respectively (Table 1). Findings for the distribution of methylation levels of HOXA9 and HIC1 in serum CFDNA of OC has been previously described (Singh et al., 2020). The methylation level of HOXA9, SOX1, and HIC1 in serum CFDNA of OC ranged from 4 to 87.4%, 4–80.6%, and 4–58.7%, respectively. However, most of the unmethylated healthy samples reflected methylation levels in the range of 0–3% (Figure 2).

DNA methylation of HOXA9, HIC1, and SOX1 was observed in 62.2% (28/45), 71.1% (32/45), and 53.3% (24/45) of the serum samples of patients with EOC. On the contrary, no promoter hypermethylation was found in serum CFDNA of control subjects for HOXA9 and HIC1. Nevertheless, SOX1 was found to be methylated in only 4.0% (1/25) of healthy control serum samples.

For all the three candidate genes, a significantly higher methylation frequency was found in serum samples of patients with OC, while in comparison to the normal controls samples (Pearson chi-square: HOXA9, *p* < 0.00001; HIC1, *p* < 0.00001 and SOX1, *p* = 0.00003, respectively).

Methylated HOXA9, HIC1, and SOX1 occurred in 62.2, 71.1, and 53.3% of OC serum samples when their promoter methylation was evaluated in the singleplex assay (AUC = 0.81, 0.88, and 0.77, respectively). The cutoff value of methylation level of all three candidate genes (HOXA9, HIC1, and SOX1), calculated using the Youden index formula, was 0.70, 0.71, and 0.62, respectively. The PPV, NPV and accuracy for these genes were, for HOXA9, 100, 59.52 and 77.10%; for HIC1, 100, 65.75 and 82.8% and for SOX1, 96.0, 53.33 and 72.8%, respectively.

**FIGURE 6:**
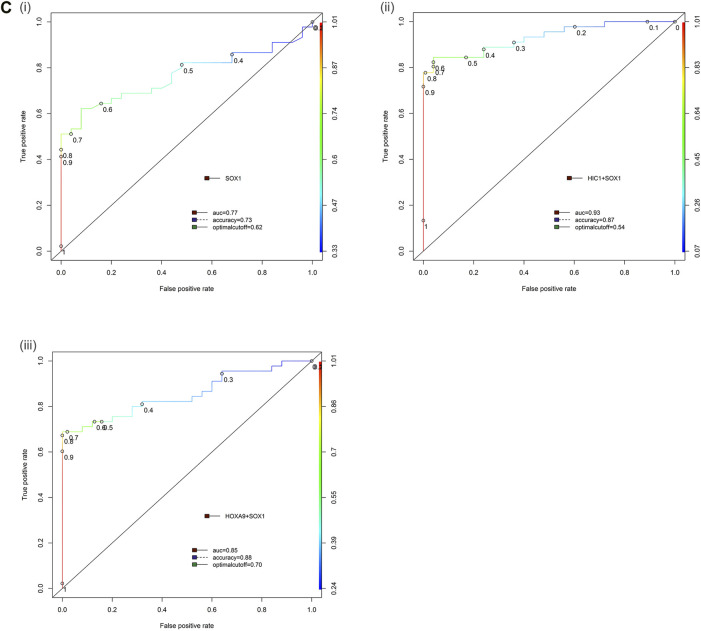
Receiver operator characteristic (ROC) curve differentiating EOC patients from healthy control in serum samples. The area under the ROC curve (AUC) suggests the accuracy of the biomarkers in distinguishing ovarian carcinoma from normal healthy control sample, is depicted for the biomarkers **(Ci)** SOX1, **(ii)** HIC1+SOX1 and **(iii)** HOXA9+SOX1 respectively. [Combined two DNA methylation marker panel] which is based on the sum of the two PMR values. AUC: area under the curve. The ROC curve for biomarker panel HOXA9+HIC1 in serum samples has been previously published (Singh et al., 2020).

In multiplex MethyLight assay, the combined methylated HIC1 + SOX1 achieved 80.0% sensitivity and 96.0% specificity with an AUC of 0.93. Simultaneous methylation of SOX1 + HOXA9 exhibited a sensitivity of 66.67% with a specificity of 96.0% (AUC = 0.85) for cancer detection in serum cell-free DNA. However, the combined sensitivity for our previously reported marker panel (HOXA9 + HIC1) was 88.9% with 100% specificity, achieving an AUC of 0.95 for differentiating serum samples of patients with EOC from normal control samples, thereby reflecting as the best discriminatory marker panel for non-invasive detection of EOC cancer using serum cell-free DNA (Table 2 and Figure 6).

We further cross-validated the performance of the best performing gene panel in discriminating cancer vs normal by data partitioning (training data set and validation data set) randomly using decision tree analysis. The performance of all the two-gene marker panels (HOXA9 + SOX1) and (HIC1 + SOX1) was found significant and the gene panels differentiated cancer vs normal with a higher accuracy in serum samples (Supplementary File 1: Table S2). The performance of two-gene marker panel HOXA9 + HIC1 in serum samples by data partitioning into training data set and test data set randomly using decision tree analysis has been previously published (Singh et al., 2020).

### Methylation Status of HOXA9, HIC1, and SOX1 in Various Stages of EOC

HOXA9, HIC1, and SOX1 were found highly positive for methylation at early stages (Stage I/II), thereby reflecting their potential to identify the early stage of the disease and serve as an early detection marker. Methylated HOXA9, HIC1, and SOX1 exhibited a similar trend to identify ovarian cancer at various stages of disease in 70.0% (7/10) patients with stage I/II cancer, 56.7% (17/30), 70.0% (21/30), and 43.3% (13/30) of stage III, respectively and 80.0% (4/5) of stage IV ovarian cancer patients.

The combined panel of HOXA9 + HIC1 and HIC1 + SOX1 exhibited the best discriminatory efficiencies at all stages of ovarian cancer (100.0% (10/10) of stage I/II, 80.0% (24/30) and 70.0% (21/30) of stage III, respectively and 80.0% (4/5) and 100.0% (5/5) of stage IV of ovarian cancer, respectively). Similarly, the two-gene panel of SOX1 + HOXA9 was found positive for methylation in 80.0% (8/10) of stage I/II, 56.7% (17/30) of stage III, and 80.0% (4/5) of stage IV ovarian cancers (Table 3).

DNA methylation status of the investigated gene promoters in OC in serum samples was independent of the tumor pathological stage and histology. No significant differences in methylation levels for these clinical features were observed. However, a significant positive association was found between the patient’s age and menopausal status with the HOXA9, HIC1, and SOX1 methylation levels in both singleplex and multiplex assay (Table 5).

**TABLE 5:** Association of promoter hypermethylation status of candidate genes with patient’s clinico-pathological features in EOC serum samples. *p*-value: Pearson’s chi-square and Fisher’s exact tests. Abbreviation: Pos: biomarker panel positive for methylation; Neg: biomarker panel negative for methylation; NS: not significant. A positive biomarker panel is defined as when either or both gene promoter show methylation.

SerumFeatures	No. of patients	HOXA9^a^			HIC1^a^		SOX1		HOXA9+HIC1^a^		HIC1+SOX1		SOX1+HOXA9	
		M	U		M	U	M	U	Pos	Neg	Pos	Neg	Pos	Neg
**Tumor**	45	28	17		32	13	24	21	40	5	36	9	30	15
**Healthy**	25	0	25		0	25	1	24	0	25	1	24	1	24
		<0.0001***			<0.0001***		< 0.0001***		<0.0001***		< 0.0001***		< 0.0001***	
**Histology**														
**Serous**	40	26		14	28	12	20	20	33	7	31	9	25	15
**Mucinous**	4	2		2	4	0	4	0	4	0	4	0	4	0
**Benign**	1	0		1	0	1	0	1	0	1	1	0	0	1
		**NS**			**NS**		**NS**		**NS**		**NS**		**NS**	
**FIGO stage**														
**I/II**	10	7		3	7	3	7	3	10	0	10	0	8	2
**III/IV**	35	21		14	25	10	17	18	28	7	26	9	21	14
		**NS**			**NS**		**NS**		**NS**		**NS**		**NS**	
**Age (Median =50)**														
**≥ Median age**	38	9		29	10	28	11	27	12	26	13	25	10	28
**< Median age**	32	19		13	22	10	14	18	26	6	20	12	20	12
		0.0033**			0.0006***		**NS**		<0.0001***		0.0299*		0.0035**	
**Menopausal status**														
**Pre**	29	14		15	17	12	13	11	21	8	17	12	15	14
**Post**	41	14		27	15	26	11	10	17	24	16	25	15	26
		**NS**			**NS**		**NS**		0.0149*		**NS**		**NS**	

a Promoter hypermethylation of DNA methylation markers-HOXA9 and HIC1 in singleplex and multiplex MethyLight assay analyzed in serum samples has been previously published (Singh et al., 2020).

## Discussion

Seven tumor suppressor genes (SOX1, SFRP1, HOXA9, RASSF1A, DAPK1, HIC1, and SPARC) were selected for the present study which has been previously reported to be extensively hypermethylated in EOC particularly serous histotype through various independent gene-specific methylation assay as well as genome-wide methylation mapping. These candidate genes are associated with various cellular functions and signaling pathways such as apoptosis (DAPK1); microtubule instability and cell cycle and mitotic arrest (RASSF1A); suppression of Wnt/β catenin signaling pathway (SFRP1 and SOX1); cell differentiation (HOXA9); cell adhesion, tumor invasion and angiogenesis (SPARC); and transcriptional repression and invasion (HIC1) (Gloss and Samimi, 2014; Grimm et al., 2019; Singh et al., 2019). Additionally, the promoter region of specified gene selected for the present study was analyzed in UCSC genome browser and the hypermethylated region in gene promoter was selected for further analysis through MethyLight and to validate the clinical significance of analyzed DMRs in the promoter region of selected genes for the diagnosis of ovarian cancer, clonal bisulfite sequencing was performed to map the consistently methylated CpGs in the gene promoter which could be responsible for downregulation of the selected genes during cancer progression.

Varying frequency of promoter hypermethylation has been reported for these seven candidate genes among various independent studies in EOC. Promoter hypermethylation of HIC1 was identified with a frequency of 17–35% in EOC tumors and was correlated with the invasive nature of the disease (Strathdee et al., 2001; Rathi et al., 2002; Teodoridis et al., 2005; Tam et al., 2007). Hypermethylation of HOXA9 was marked preferentially in early-stage epithelial ovarian cancer tumors (methylation frequency 51%) and in endometrioid subtype, strongly underlining its association with increased risk of EOC (Wu et al., 2007; Widschwendter et al., 2009). RASSF1A was detected to be methylated in EOC tumors with a frequency of 15–50% and was associated with the presence of malignancy (Yoon et al., 2001; Makarla et al., 2005; Choi et al., 2006; Bol et al., 2010). Similarly, Socha et al. reported hypermethylation of SPARC in 68% of EOC tumors with high methylation exhibited by high-grade serous EOC (Socha et al., 2009). DAPK1 was reported to be methylated in EOC tumors with a frequency of 50–67%, and its silencing induced by promoter methylation was correlated with metastatic disease (Collins et al., 2006; Häfner et al., 2011). Methylation of SFRP1 was identified with a frequency of 5–35% in epithelial ovarian cancer and was correlated with survival (Takada et al., 2004; Teodoridis et al., 2005; Su et al., 2009). Su et al. reported SOX1 methylation in 58.7% of EOC tumors and was correlated with overall survival (Su et al., 2009). The majority of these studies were analyzed by the MSP-PCR technique in tissue samples. However, no single report has examined the simultaneous methylation of the selected tumor suppressor genes/genes panel by MethyLight assay in tissue or any body fluids.

Considering previously published reports on gene promoter hypermethylation in EOC, we aimed to define the most specific gene panel for EOC detection in tissue and cell-free DNA samples. Herein, we report the exploration of potential genes for their significant diagnostic relevance as a minimally invasive methylation-based biomarker and their utility to construct a multiplex MethyLight assay to access the feasibility of concurrent analysis of the promoter methylation status of candidate gene/gene panels in tissue and serum cell-free DNA. Numerous reports based on quantitative methylation analysis of gene promoter using MethyLight assay have confirmed its high sensitivity for detecting methylated alleles and its potential for clinical applications (He et al., 2010; Lind et al., 2011; Warren et al., 2011; Andresen et al., 2015). The feasibility of analyzing multiple loci simultaneously in a single assay along with the minute utility of template DNA makes the multiplex MethyLight assay an excellent fit for analysis of clinical samples with minimal DNA amount, such as when working with liquid biopsies. We estimated the sensitivity, specificity, and reliability of detection of the multiplex MethyLight assay, along with interassay variability. The proposed assay exhibited high sensitivity of detection and good reproducibility, as indicated by the linearity and accuracy of the MethyLight assay. A strong correlation was observed between the normalized ratio of a singleplex and multiplex assay for all the candidate genes under study in tissue and serum samples, reflecting equal performance and higher sensitivity of the assay.

We evaluated the diagnostic performance of the promoter hypermethylation of individual genes and gene panels in tissue samples for early detection of EOC. Our findings have confirmed the aberrant promoter hypermethylation status of these tumor suppressor genes when analyzed in singleplex MethyLight assay in tissue samples from ovarian cancer patients, which is in accordance with the previously published studies. In addition, our findings showed that the methylation levels of candidate genes (HIC1, HOXA9, SOX1, DAPK1, RASSF1A, SFRP1, and SPARC) were significantly elevated in patients with ovarian cancer and was highly cancer-specific, which is in concord with the previously published reports. Moreover, higher sensitivity in identifying early stages of ovarian cancer was exhibited by some of the two-gene combination marker panels, thereby reflecting their potential to serve as early detection marker. Our result also suggests that the methylation of these seven candidate genes might be a common epigenetic alternation associated with the development and progression of epithelial ovarian cancer and could also be involved in the early stages of ovarian cancer tumorigenesis.

The performance of these genes excluding SPARC (which disclosed the lowest sensitivity and specificity) was further analyzed through multiplex MethyLight assay to select the best performing gene cassette for further evaluation of their promoter methylation in CFDNA. The possible two gene combinations (HOXA9+HIC1, SOX1+HIC1, HIC1+SFRP1, RASSF1A + HOXA9, DAPK1+SOX1, and HOXA9+SOX1) were able to provide a high sensitivity varying in a range from 73.0–88.0% and specificity in a range from 77–89%. Considering sensitivity, specificity, and accuracy to identify disease, the best performing gene panels in EOC tissue samples were a two-gene combination of HOXA9+HIC1, HOXA9+SOX1, HIC1+SOX1. Our previously identified two gene-marker panel of HOXA9+HIC1 attained a sensitivity of 88.2% and specificity of 88.57% [AUC = 0.92] when either or both genes showed promoter methylation in tissues samples (Singh et al., 2020). The 2-gene combination (HOXA9+SOX1 or SOX1+HIC1) exhibited similar specificity and almost equivalent sensitivity for EOC detection compared to other gene methylation marker panels evaluated in the present study. Owing to this, to investigate the diagnostic relevance as a non-invasive biomarker for EOC, these genes were screened for further validation of their methylation status in serum DNA. To the best of our knowledge, this study reports for the first time the concurrent promoter hypermethylation of these gene panels in tissue samples of EOC patients through multiplex qPCR assay. We also performed qRT-PCR for some of the tumor samples (18 malignant and 10 controls) to evaluate the expression profiling of candidate genes used in the present study. Here, we found that HIC1, DAPK1, HOXA9 and SOX1 were downregulated while RASSF1A was found marginally upregulated (Supplementary File 1: Figure S6).

Relatively few studies evaluating concurrent methylation of multiple genes have been reported in EOC. For instance, Melnikov et al. using a microarray-based technique identified -10 potentially informative genes in tissue samples, which demonstrated a maximum sensitivity and specificity of 69 and 70%, respectively, in various gene combinations, indicating the presence of cancer. However, the clinical relevance of this panel towards early-stage detection of EOC is less understood, as all advanced-stage tumors (either stage IIIA or higher) were analyzed in the study (Melnikov et al., 2009). Similarly, another study highlighted that promoter methylation of at least one of the six genes in the panel (BRCA1, p16ink4a, APC, p14arf, RASSF1A, and DAPK) could efficiently differentiate patients with ovarian cancer from healthy control with 99% (70/71) sensitivity and 100% specificity, utilizing multiplex methylation-specific PCR (MS-PCR) (de Caceres et al., 2004). However, the major limitation to conclude the clinical significance of the reported finding was the sample size and the sensitivity of the technique used for the study. Su et al. demonstrated that hypermethylation of any one of SOX1, PAX1, and SFRP1 genes exhibited 73.08% sensitivity with a specificity of 75% in discriminating malignant ovarian cancer samples from healthy controls. The diagnostic relevance of this marker panel as a potential test for OC detection cannot be underlined as neither the test score are probably high enough (though higher than CA125 alone), nor any specification with respect to tumor stage within the studied group was furnished (Su et al., 2009).

Considering non-invasive detection of ovarian cancer, relatively very few studies have highlighted analyses of the methylation status of potential genes, which might serve as a biomarker in serum/plasma samples. For instance, DAPK promoter hypermethylation in peripheral blood achieved 54% (14/16) sensitivity and 100% ((10/10) specificity using methylation-specific PCR (MS-PCR) (Collins et al., 2006). Similarly, in another study by Melnikov et al., five potentially informative genes were identified in plasma samples using a Microarray-based multiplex assay which achieved a maximum sensitivity and specificity of 61 and 85%, respectively to indicate the presence of OC (Melnikov et al., 2009). Promoter hypermethylation of RASSF1A and BRCA in serum CFDNA could signify the presence of ovarian cancer with a sensitivity and specificity of 82 and 100%, respectively, using MS-PCR (de Caceres et al., 2004). Another study by Zhang et al. showed that the hypermethylation of at least one of seven gene panel, which includes RUNX3, APC, CDH1, TFPI2, RASSF1A, SFRP5, and OPCML, in serum cell-free DNA could be detected with sensitivity and specificity of 85.3 and 90.5% respectively, in stage I EOC using a novel multiplex MS-PCR assay. The detection figures achieved using this assay were noticeably higher than CA125 alone, which reflected a sensitivity and specificity of 56.1 and 64.15%, respectively (Zhang et al., 2013). The majority of the previously reported studies have utilized MS-PCR to analyze gene promoter hypermethylation which facilitates a “yes” or “no” answer to reflect the presence of malignancy.

We evaluated and validated the promoter methylation status of the best performing genes (HIC1, HOXA9, and SOX1) in serum CFDNA of 70 matched EOC samples. The promoter hypermethylation status of HOXA9 and HIC1 in serum CFDNA has been previously published (Singh et al., 2020). Our finding confirms promoter hypermethylation of these genes in cell-free DNA, which was detected more frequently in serum of cancer patients than healthy controls. In accordance with other published studies, a lower methylation level was observed in circulating serum cell-free DNA compared to the tissue tumor samples. The considerably minute amount of CFDNA, its loss during extraction and sodium bisulfite conversion step, and the high background of normal DNA could be the plausible rationale for this. Individual genes (HOXA9, HIC1, and SOX1) and all possible 2-gene combination marker panels were highly positive for methylation at early stages (Stage I/II), thereby reflecting their potential to identify the early stage of the disease and could serve as an early detection marker.

The possible two gene combinations (HOXA9+HIC1, SOX1+HIC1, HOXA9+SOX1) provided a high sensitivity varying in a range from 67.0–89.0% and specificity in a range from 96–100% in serum CFDNA. Considering sensitivity, specificity, and accuracy to identify disease, the best discriminatory gene panel in EOC serum samples was the two-gene combination of HOXA9+HIC1, which has been previously published (Singh et al., 2020). The other two-gene cassette comprising of SOX1+HIC1 exhibited combined sensitivity of 80.0% with a specificity of 96% in serum CFDNA [AUC = 0.93] in differentiating malignant EOC samples and cancer-free healthy control serum samples. The two-gene combination of SOX1 and HOXA9 also attained similar specificity [AUC = 0.85]. These results highlight the enhanced diagnostic potential of methylated markers in serum CFDNA from epithelial ovarian cancer patients. To the best of our knowledge, this study reports for the first time the concurrent promoter hypermethylation of these gene panels in serum samples of EOC patients through multiplex qPCR assay. Furthermore, we assessed the performance of the candidate marker panel along with clinical predictor CA125 to evaluate the accuracy of prediction of ovarian cancer. Tissue biopsies recruited for the present study were histologically verified and confirmed for their malignant status. Elevated CA125 was not significant in discriminating 16 malignant cases. However, the candidate marker panels performed better in discriminating these samples as malignant (HIC + SOX [n = 7/16]; HOXA9+HIC1 [n = 12/16]; SOX + HOXA9 [*n* = 6/16]). In addition, the gene panels combined with CA125 exhibited improved diagnostic accuracy in discriminating cancer from normal samples as discussed in (Supplementary File 1: Figure S5 and Table S4).

Furthermore, the quantitative evaluation of gene promoter methylation through MethyLight assay was confirmed and validated by clonal bisulfite sequencing of selected tumor tissue and normal DNA samples for HOXA9, HIC1 (Singh et al., 2020), and SOX1 gene. The methylation density in the tumor sample was substantially higher, especially in the region assessed by MethyLight assay in contrast to their normal counterpart, where no hypermethylation was observed. The results obtained from the MethyLight assay exhibited high concordance with the clonal bisulfite sequencing data, thereby strongly supporting the highly sensitive detection of ovarian cancer through multiplex MethyLight assay.

As shown in Table 1, the median PMR values of tumor tissues and serum exhibited significant differences when compared with tissues and serum obtained from healthy subjects. As listed in Tables 4, 5, no significant association could be observed between gene/gene panel promoter methylation level and CA125 level, histology, or FIGO stage. However, a significant positive correlation between candidate gene promoter methylation and age and menopausal status of patients was observed.

Our data confirm the high diagnostic performance of the novel epigenetic marker panels constituting of SOX1, HOXA9, and HIC1 gene in various combinations, in matched tissue and serum samples from EOC patients. Overall, our study strongly highlights the diagnostic relevance of serum DNA methylation markers in predicting the presence of EOC. However, further validation of our finding in a larger cohort still needs to be investigated. Two significant limitations of the present study are the limited sample size and the lack of long-term follow-up, especially of the asymptomatic controls, which tested positive and could probably develop ovarian cancer. Moreover, we could not establish any association between the concurrent hypermethylation of combined gene panels with disease prognosis or presence of residual disease or resistance to drug or recurrence or overall survival rate, etc., due to lack of relevant follow-up patient information and ongoing treatment. Moreover, higher sensitivity in identifying early stages of ovarian cancer was exhibited by HOXA9, HIC1, and SOX1, which further confirms their utility as a potential biomarker for early-stage detection of EOC. Furthermore, the study has provided a “Proof of concept” that highlights the feasibility of multiplex assay and the potential of epigenetic signatures to develop an effective non-invasive biomarker panel for ovarian cancer diagnostics.

The previously published report of multiplexing two-gene (HOXA9 and HIC1) aims at the development of an assay based on DNA methylation signatures as biomarkers for EOC detection at an early stage. Moreover, the present study primarily highlights the exploration of potential genes for their significant diagnostic relevance (sensitivity and specificity) as a minimally invasive epigenetic-based biomarker, and further implicates that multiplexing of more than two genes could be of great significance towards the development of a more sensitive and specific assay in future. Considering the enhanced performance of this multiplex MethyLight assay, the study may further be taken forward for evaluating the clinical utility of SOX1, HOXA9, and HIC1 genes as DNA methylation-based biomarkers, in a larger pool of serum samples, particularly of early-stage (I/II) in a multi-centric approach. Further, evaluation and validation of the diagnostic performance of triple gene combination marker panel (HOXA9+HIC1+SOX1) alone or along with some novel biomarkers identified from integrated methylome analysis might hold great promise to facilitate the basis for an early-stage diagnostic assay for EOC screening. This method is highly specific, sensitive, and reproducible; moreover, it also facilitates rapid and accurate detection of biologically significant information taking into the utility of minute amounts of modified DNA in patient blood samples. This multiplex MethyLight assay holds the potential to serve as an efficient and reliable tool in clinical ovarian cancer blood-based testing in the future.

## Conclusion

The present study would lead to the utilization of specific methylation signatures through multiplex MethyLight assay towards developing an effective non-invasive tool for early detection of ovarian cancer using serum/plasma as a sample. The findings further underline highly sensitive and specific minimally invasive multiplex-methylation-based test (based on concurrent methylation analysis), which might improve patient compliance, increase tumor diagnosis at an earlier stage, and would be valuable in significantly reducing ovarian cancer-associated morbidity and mortality. Nevertheless, the potential utility of these methylation-based biomarkers requires further validation in a larger cohort of EOC suspects to develop a minimally-invasive methylation-based test for accurate detection of clinically significant EOC.

## Data Availability

The original contributions presented in the study are included in the article/Supplementary Material, further inquiries can be directed to the corresponding author.

## Abbreviation

HIC1, hypermethylated in cancer 1; HOXA9, homeobox gene A9; DAPK1, death associated protein kinase 1; RASSF1A, ras association domain family 1 isoform A; SFRP1, secreted frizzled-related protein 1; SOX1, SRY-Box Transcription Factor 1; SPARC, secreted protein, acidic and rich in cysteine; CFDNA, cell free DNA; EOC, epithelial ovarian cancer; OC, ovarian cancer; ROC, receiver operator characteristics curve; AUC, area under the curve; PMR, percentage of methylated reference; PPV, positive predictive value; NPV, negative predictive value; Pos, biomarker panel positive for methylation; Neg, biomarker panel negative for methylation; NS, not significant; R^2^, correlation coefficient; MS-PCR, methylation-specific PCR; CA125, cancer antigen-125; FFPE, formalin-fixed, paraffin-embedded.
