# Supplementary material for: Evaluation of the Diagnostic Potential of Candidate Hypermethylated Genes in Epithelial Ovarian Cancer in North Indian Population
Source: Front Mol Biosci. 2021 Oct 28;8:719056. doi: 10.3389/fmolb.2021.719056 (PMC8581490; doi:10.3389/fmolb.2021.719056)
Supplement: Supplementary file 1 [file DataSheet1.docx]

**Supplementary file 1:**

- **Supplementary Figures:**

1. **Figure S1: MethyLight assay development.** The standard curves of **(a)** COL2A1, **(b)** SOX1, **(c)** DAPK1, **(d)** SPARC, **(e)** RASSF1A and **(f)** SFRP1 generated with methylation positive MSss1-treated DNA in a multiplex PCR. The Correlation coefficients (R²) for the test of a linear association were 0.9952, 0.9628, 0.9963, 0.9699, 0.9341 and 0.9790, respectively. Standard curves for HOXA9 and HIC1 gene has been previously published (Singh et al., 2020)**.**
2. **Figure S2:** Amplification plots of the candidate biomarker genes and the control gene COL2A1, using singleplex **(i) (iii) (v) (vii) (ix) & (xi)** and multiplex **(ii) (iv) (vi) (viii) & (x)** MethyLight assays in tissue samples **(A)** respectively. Amplification plots of the best performing genes (HOXA9, HIC1, SOX1) narrowed down for further validation in serum samples **(B)** using singleplex **(i)** and multiplex **(ii)** & **(iii)** MethyLight assays respectively. ΔRn is defined as the cycle-to-cycle change in the reporter fluorescence signal normalized to a passive reference fluorescence signal. Amplification plots of the candidate biomarker genes HIC1 and HOXA9, using singleplex and multiplex MethyLight assays in tissue and serum samples respectively has been previously published (Singh et al., 2020)**.**
3. **Figure S3:** Correlation between the ΔCq values obtained with singleplex and multiplex assays for **(A) (i) (ii) & (iii)** SOX1 (multiplexed with HOXA9, HIC1 and DAPK1, respectively), **(iv)** DAPK1, **(v)** RASSF1A, **(vi)** SFRP1, **(vii) & (viii)** HIC1 (multiplexed with SFRP1 and SOX1, respectively) and **(ix) & (x)** HOXA9 (multiplexed with RASSF1A and SOX1, respectively) genes, respectively in tissue samples. The ΔCq values between singleplex and multiplex MethyLight assay differ due to differences in set thresholds. The normalized ratios of singleplex and multiplex MethyLight assay for HOXA9 (multiplexed with HIC1) and HIC1 (multiplexed with HOXA9) in tissue samples has been previously published (Singh et al., 2020)**.**
4. **Figure S4:** **Receiver operator characteristic (ROC) curve differentiating EOC patients from healthy control in tissue samples**. The area under the ROC curve (AUC) suggests the accuracy of the biomarkers in distinguishing ovarian carcinoma from normal healthy control sample, is depicted for the biomarkers **(A)** **(i)** SOX1, **(ii)** DAPK1, **(iii)** RASSF1A **(iv)** SFRP1 and **(v)** SPARC, respectively. AUC: area under the curve. The ROC curve for biomarker HOXA9 and HIC1 in tissue samples has been previously published (Singh et al., 2020)**.**
5. **Figure S5: Receiver-operating characteristic (ROC) curves accessing the accuracy of predictions for ovarian cancer using the candidate gene marker panel along with clinical predictor (CA125) in serum.**
6. **Figure S6: Expression analysis of candidate genes-RASSF1A, HIC1, DAPK1, HOXA9 and SOX1 through Real-time quantitative PCR (RT-qPCR). Log2FC values were used to compare the expression of genes between malignant and control samples.**

- **Supplementary Figures**

1. **Figure S1:
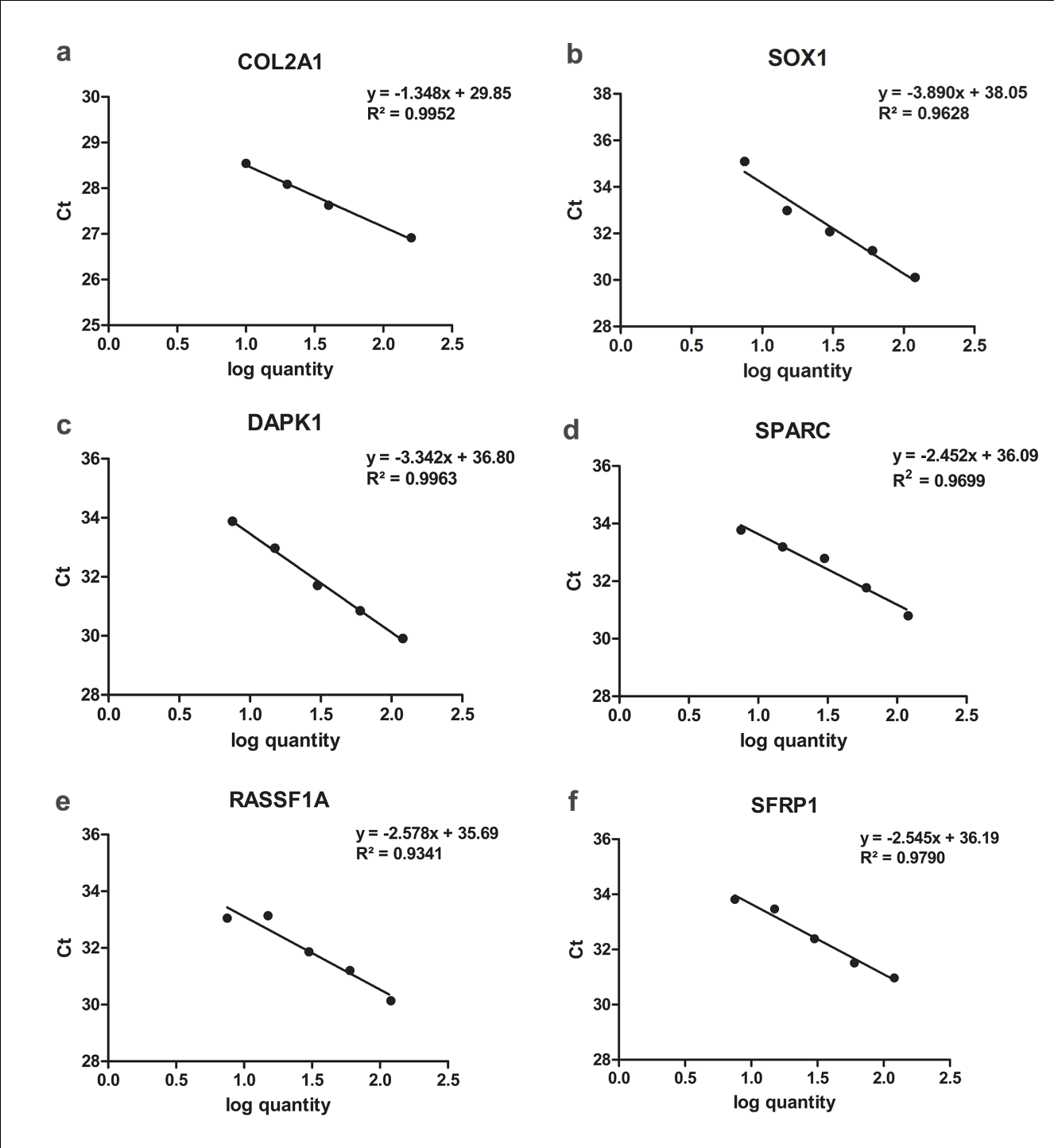
**

**Figure S1: MethyLight assay development.** The standard curves of **(a)** COL2A1, **(b)** SOX1, **(c)** DAPK1, **(d)** SPARC, **(e)** RASSF1A and **(f)** SFRP1 generated with methylation positive MSss1-treated DNA in a multiplex PCR. The Correlation coefficients (R²) for the test of a linear association were 0.9952, 0.9628, 0.9963, 0.9699, 0.9341 and 0.9790, respectively. Standard curves for HOXA9 and HIC1 gene has been previously published (Singh et al., 2020)**.**

1. **Figure S2:**

**
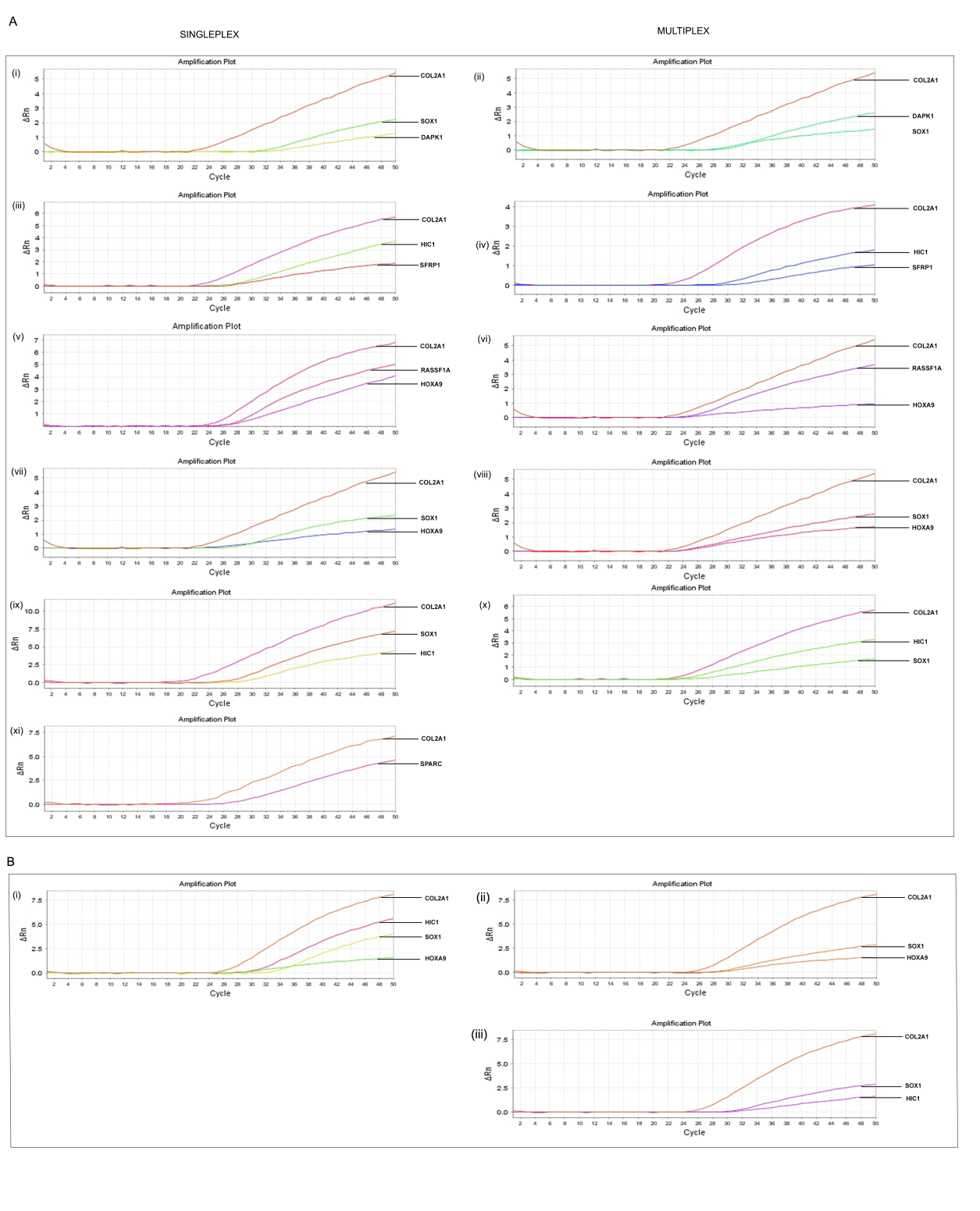
**

**Figure S2:** Amplification plots of the candidate biomarker genes and the control gene COL2A1, using singleplex **(i) (iii) (v) (vii) (ix) & (xi)** and multiplex **(ii) (iv) (vi) (viii) & (x)** MethyLight assays in tissue samples **(A)** respectively. Amplification plots of the best performing genes (HOXA9, HIC1, SOX1) narrowed down for further validation in serum samples **(B)** using singleplex **(i)** and multiplex **(ii)** & **(iii)** MethyLight assays respectively. ΔRn is defined as the cycle-to-cycle change in the reporter fluorescence signal normalized to a passive reference fluorescence signal. Amplification plots of the candidate biomarker genes HIC1 and HOXA9, using singleplex and multiplex MethyLight assays in tissue and serum samples respectively has been previously published (Singh et al., 2020)**.**

1. **Figure S3:**

**
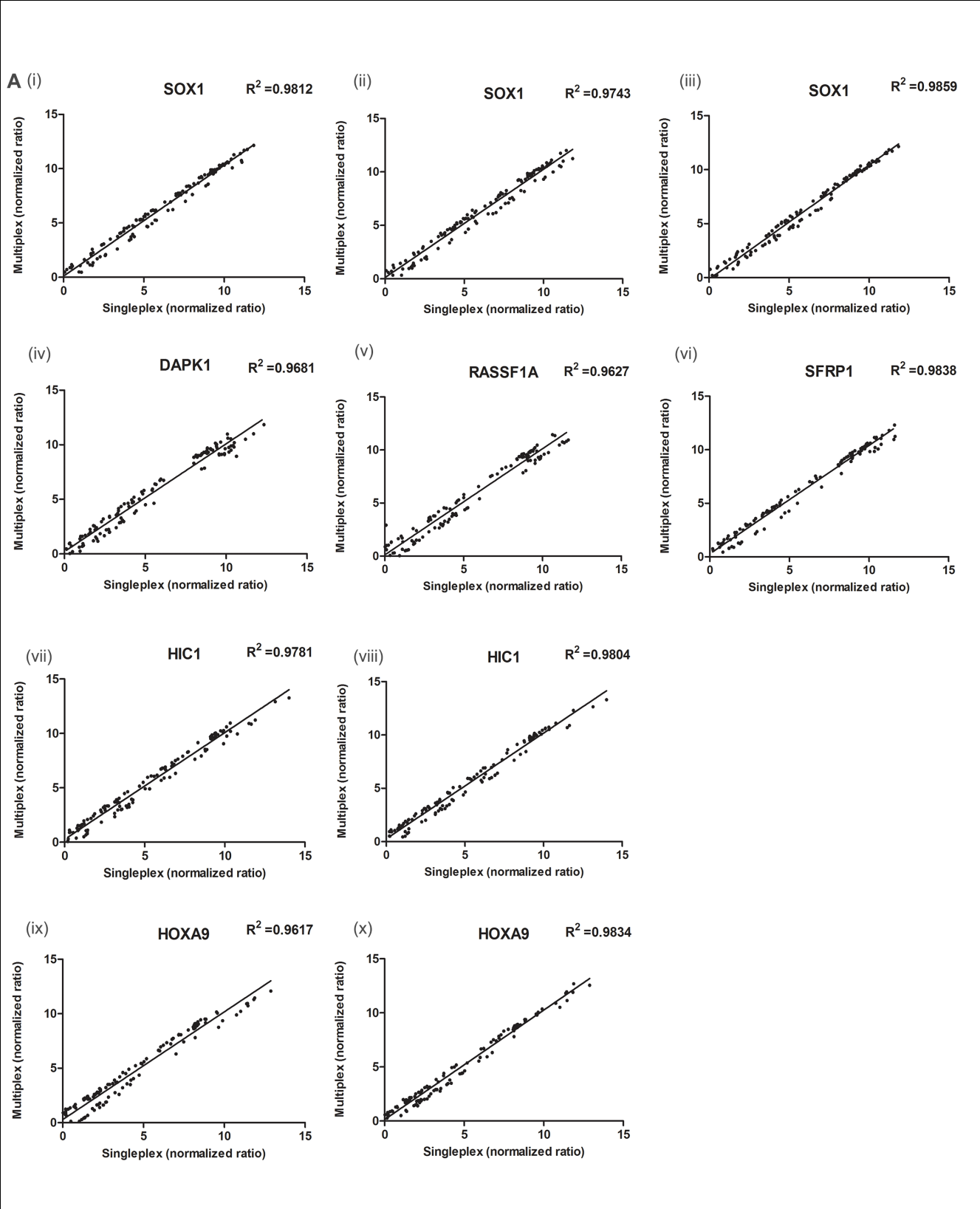
**

**Figure S3:** Correlation between the ΔCq values obtained with singleplex and multiplex assays for **(A) (i) (ii) & (iii)** SOX1 (multiplexed with HOXA9, HIC1 and DAPK1, respectively), **(iv)** DAPK1, **(v)** RASSF1A, **(vi)** SFRP1, **(vii) & (viii)** HIC1 (multiplexed with SFRP1 and SOX1, respectively) and **(ix) & (x)** HOXA9 (multiplexed with RASSF1A and SOX1, respectively) genes, respectively in tissue samples. The ΔCq values between singleplex and multiplex MethyLight assay differ due to differences in set thresholds. The normalized ratios of singleplex and multiplex MethyLight assay for HOXA9 (multiplexed with HIC1) and HIC1 (multiplexed with HOXA9) in tissue samples has been previously published (Singh et al., 2020)**.**

1. **Figure S4:**

**
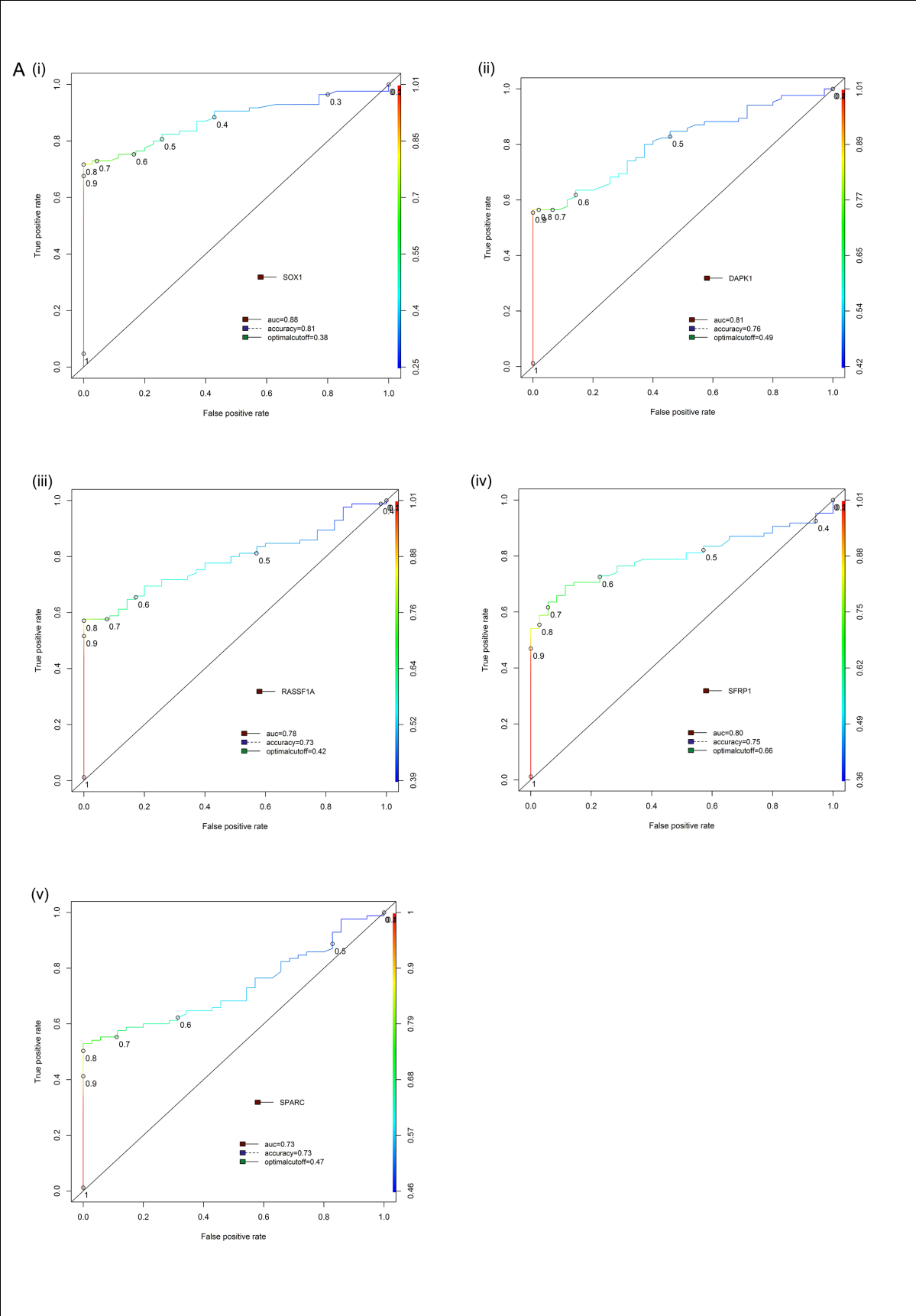
**

**Figure S4:** **Receiver operator characteristic (ROC) curve differentiating EOC patients from healthy control in tissue samples**. The area under the ROC curve (AUC) suggests the accuracy of the biomarkers in distinguishing ovarian carcinoma from normal healthy control sample, is depicted for the biomarkers **(A)** **(i)** SOX1, **(ii)** DAPK1, **(iii)** RASSF1A **(iv)** SFRP1 and **(v)** SPARC, respectively. AUC: area under the curve. The ROC curve for biomarker HOXA9 and HIC1 in tissue samples has been previously published (Singh et al., 2020)**.**

**5. Figure S5:**

**
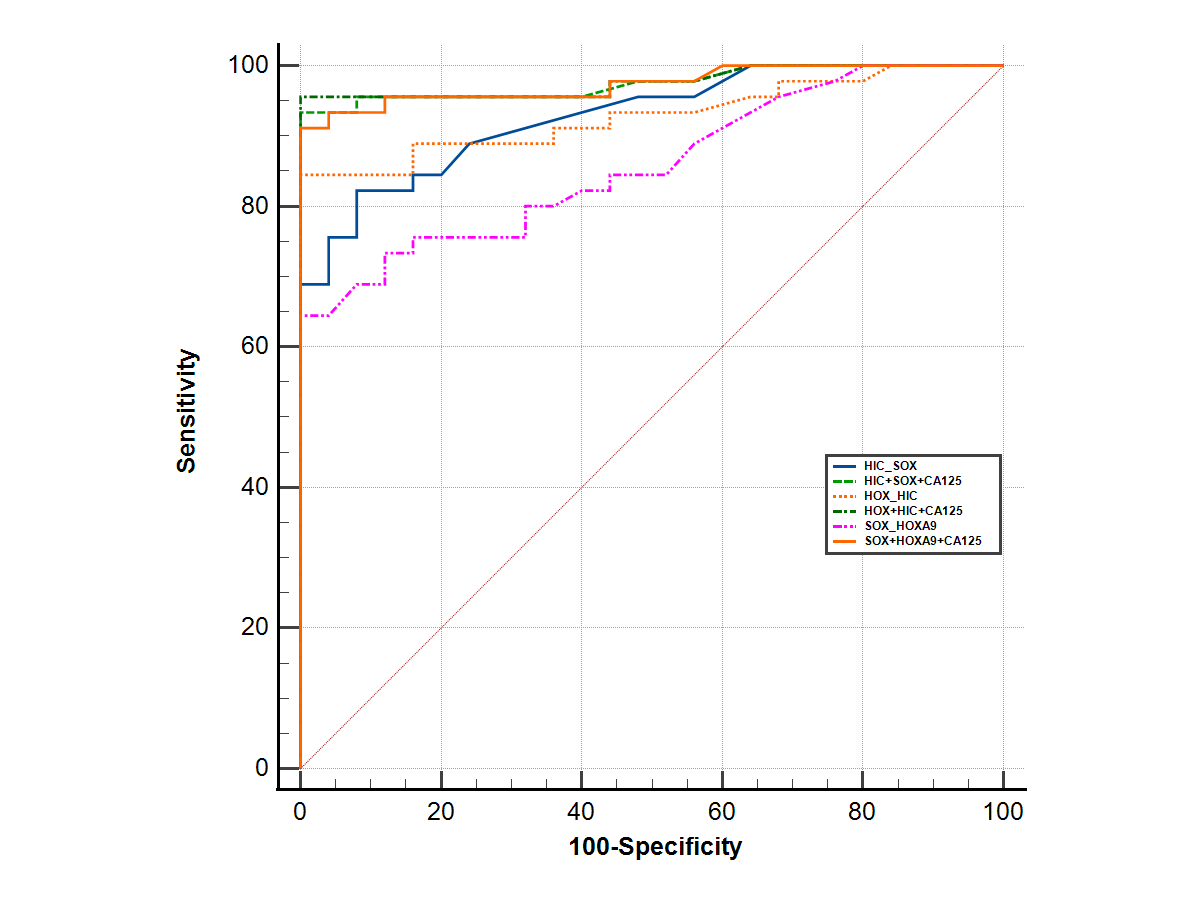
**

**Figure S5: Receiver-operating characteristic (ROC) curves accessing the accuracy of predictions for ovarian cancer using the candidate gene marker panel along with clinical predictor (CA125) in serum.**

**6. Figure S6:**

**
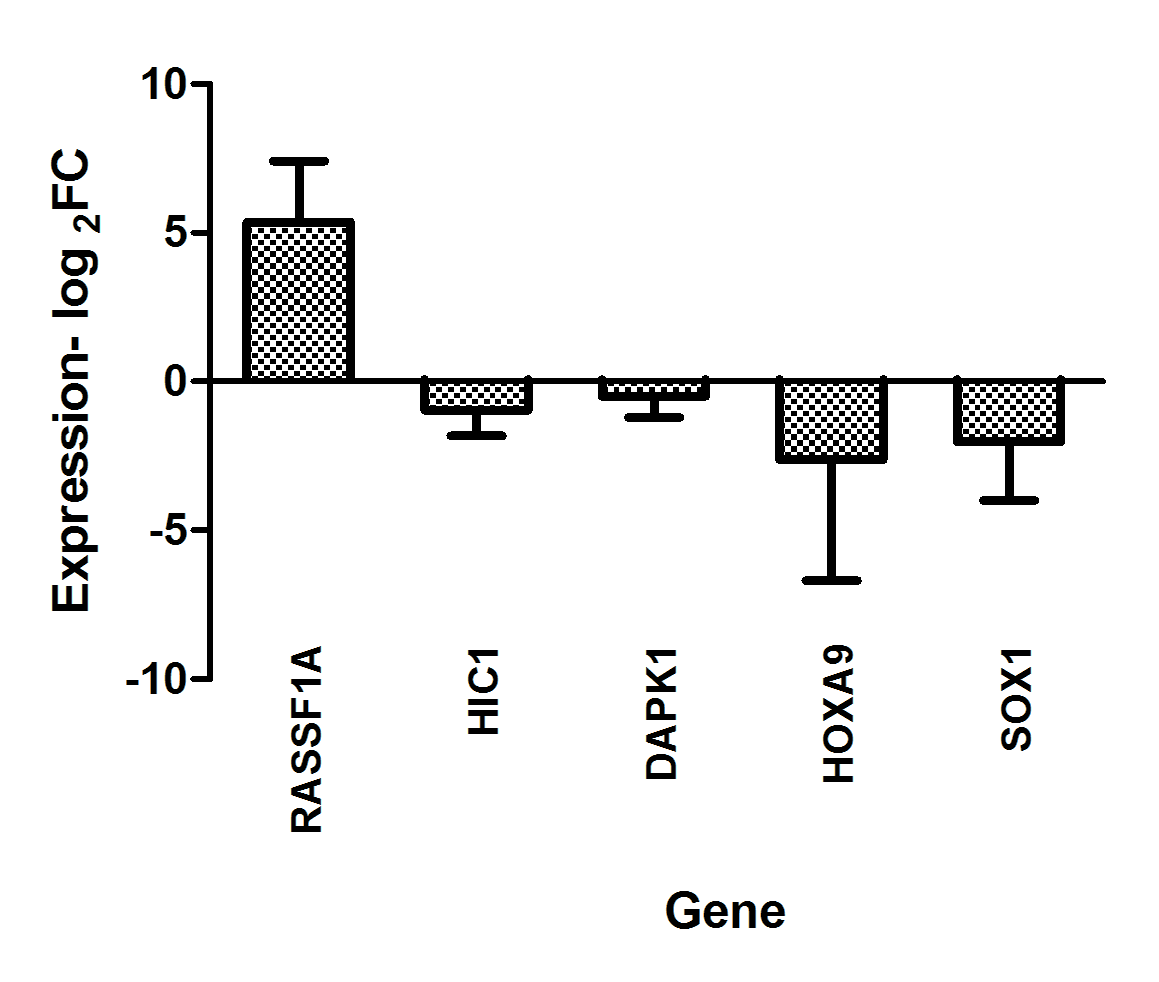
**

**Figure S6: Expression analysis of candidate genes-RASSF1A, HIC1, DAPK1, HOXA9 and SOX1 through Real-time quantitative PCR (RT-qPCR). Log2FC values were used to compare the expression of genes between malignant and control samples.**

- **Supplementary Tables:**

**Table S1: Primers used for (a) quantitative Real time PCR Reaction (MethyLight) and (b) clonal bisulfite sequencing**. Amplicon annotation lists the start and end point of each amplicon relative to the transcription start point (in bases) provided by the USCS Genome Browser <http://genome.ucsc.edu/>. (Degeneration: Y=C/T, R=G/A)**.** Primers and probe for the HOXA9 and HIC1 MethyLight assay and clonal bisulfite sequencing have been described previously (Singh et al., 2020)**.**

1. **MethyLight Primers and Probe Sequences**

| **HUGO Gene Nomen-clature** | **Chromo-somal location** | **Amplicon annotation** | **location relative to transcription start (bp)** | **TSS Annotation** | **Length** | **Forward Primer Sequence 5’-3’** | **Reverse Primer Sequence 5’-3’** | **Probe Oligo Sequence 5’-3’** | **No. of CpGs** |
| --- | --- | --- | --- | --- | --- | --- | --- | --- | --- |
| **COL2A1** | chr12 (q13.11) | 47,987,446 - 47,987,537 | - | - | 92 | TCTAACAATTATAAACTCCAACCACCAA | GGGAAGATGGGATAGAAGGGAATAT | 5’-6-FAM-CCTTCATTCTAA  CCCAATACCTATCCCACCTCTAAA-TAMRA-3’ | - |
| **HIC1** | Chr17 (p13.3) | 2056615-2056716 | -55/+45 | 2056671 | 101 | GTTAGGCGGTTAGGGCGTC | CCGAACGCCTCCATCGTAT | 5’-6-FAM-CAACATCGTCTA  CCCAACACACTCTCCTACG-TAMRA-3’ | 11 |
| **HOXA9** | Chr7 (p15.2) | 27166222-27166334 | -692/-804 | 27165530 | 112 | AAAATCCGTCCCAAACGAAA | CGTTTTAGGCGGGTAGTTGTG | 5’-VIC-CGCCGACCCTACCA  AAACACTCCAA-TAMRA-3’ | 10 |
| **RASSF1A** | Chr3 (p21.31) | 50340720-50340784 | -152/-217 | 50340936 | 65 | ATTGAGTTGCGGGAGTTGGT | ACACGCTCCAACCGAATACG | 5’-6-FAM-CCCTTCCCAACG  CGCCCA-TAMRA-3’ | 7 |
| **SFRP1** | chr8 (p11.21) | 41,309,538 -41,309,591 | -21/-94 | 41,309,498 | 73 | CAACTCCCGACGAAACGAA | CGCGAGGGAGGCGATT | 5’-VIC-CACTCGTTACCACG  TCCGTCACCG-TAMRA-3’ | 13 |
| **SPARC** | chr5 (q33.1) | 151,686,858 -151,686,929 | + 235/+307 | 151,687,165 | 72 | TTTCGCGGTTTTTTAGATTGTTC | CATACCTCAATAACAAACAAACAAACG | 5’-6-FAM-CAAAACGCGCT  CTC-MGB-Eclipse®-3’ | 6 |
| **SOX1** | chr13(q34) | 112,067,216-112,067,290 | -355/-430 | 112067646 | 75 | AACCCGACCCGAAATATACTATCTC | GAGGATCGAGCGTAGGAGGAA | 5’-VIC-CGACCGCCGCTACG  CGCTATCT- TAMRA-3’ | 10 |
| **DAPK1** | Chr9 (q21.33) | 87,498,016 – 87,498,083 | -885/-953 | 87,498,969 | 68 | TCGTCGTCGTTTCGGTTAGTT | TCCCTCCGAAACGCTATCG | 5’-6-FAM-CGACCATAAAC  GCCAACGCCG- TAMRA-3’ | 11 |

**(b) Clonal Bisulfite sequencing primers**

| **Gene name** | **Amplicon location relative to transcription start (bp)** | **No. of CpGs** | **Product Size (bp)** | **Forward Primer Sequence 5’-3’** | **Reverse Primer Sequence 5’-3’** | **Tm (°C)** |
| --- | --- | --- | --- | --- | --- | --- |
| **HIC1** | -49/+84 | 14 | 176 | AAGTGTYGGGTTGGGGTTAG | TCRCACAAAAAACCCTTAATA | 54 |
| **HOXA9** | -604 /- 801 | 19 | 197 | TTTAGGYGGGTAGTTGTGGGG | AATTAACRAACTCCCTATAAATAATCCC | 60 |
| **SOX1** | -207 /- 423 | 26 | 216 | GAGYGTAGGAGGAAGGAGATAG | ATACACAAACCACTTACCAAAAAAACC | 60 |

**Table S2:** **Data partitioning and validation of model performance of two-gene panels in tissue and serum samples.** The performance of two-gene marker panel HOXA9 and HIC1 in tissue and serum samples by data partitioning into training data set and test data set randomly using decision tree analysis has been previously published (Singh et al., 2020).

| **Tissue** | | | | |
| --- | --- | --- | --- | --- |
|  | | **Training** | **Validation** | **P value** |
| **HIC1+SOX1** | **Accuracy** | 0.843 | 0.833 | 1.392929e-15  Significant |
|  | **Misclassification error** | 0.157 | 0.167 |  |
| **SOX1+HOXA9** | **Accuracy** | 0.863 | 0.778 | 6.40562e-17  Significant |
|  | **Misclassification error** | 0.137 | 0.222 |  |
| **HOXA9+RASSF1A** | **Accuracy** | 0.814 | 0.667 | 2.194107e-13 Significant |
|  | **Misclassification error** | 0.186 | 0.333 |  |
| **HIC1+SFRP1** | **Accuracy** | 0.794 | 0.722 | 2.608936e-13 Significant |
|  | **Misclassification error** | 0.206 | 0.278 |  |
| **DAPK1+SOX1** | **Accuracy** | 0.745 | 0.778 | 9.161025e-12 Significant |
|  | **Misclassification error** | 0.255 | 0.222 |  |
| **Serum** | | | | |
| **HIC1+SOX1** | **Accuracy** | 0.833 | 0.9 | 1.856965e-10  Significant |
|  | **Misclassification error** | 0.167 | 0.1 |  |
| **SOX1+HOXA9** | **Accuracy** | 0.783 | 0.8 | 3.488409e-08 Significant |
|  | **Misclassification error** | 0.217 | 0.2 |  |

**Table S3: Clinical and pathological features of the study cohort: (a) Malignant samples and (b) Normal control samples.**

1. **Malignant samples**

| **Patient ID No.** | **Age/Sex** | **Menopausal Status** | **Abdomen Distention** | **Size of Tumor** | **Lt/Rt** | **Mobile** | **Consistency Cystic/variegated/solid/any other** | **CA125 Level** | **Histotype** | **Benign** | **Malignant** | **FIGO- stage** |
| --- | --- | --- | --- | --- | --- | --- | --- | --- | --- | --- | --- | --- |
| 1 | 40/F | Post | No | 6.6 cm(L) 4.3 cm(R) | B/L | Restricted mobility | Variegated | 33 | Serous Papillary Adeno Ca | No | Yes | III |
| 2 | 45/F | Post | - | 4.3 cm |  | Yes | Variegated | 0.17 | Serous Papillary Adeno Ca | No | Yes | III |
| 3 | 55/F | Post | No | 3.7 cm | Lt | Yes | Variegated | 65.7 | Serous Adeno Ca | No | Yes | III |
| 4 | 40/F | Pre | Yes | 7 cm | B/L | Yes | Variegated | 762 | Serous Adeno Ca | No | Yes | III |
| 5 | 45/F | Post | No | 5.5 cm | Lt | Yes | Variegated | 57 | Serous Adeno Ca | No | Yes | III |
| 6 | 55/F | Post | No | 4.0 cm | Rt | Yes | Cystic | 5 | Serous Adeno Ca | No | Yes | III |
| 7 | 30/F | Pre | No | 5.0 cm | Lt | No | Cystic | 134 | Serous | No | Yes | IIIC |
| 8 | 50/F | Post | No | 15.0 cm | Rt | Yes | Variegated | 116 | Serous | No | Yes | IIIC |
| 9 | 50/F | Post | No | 20x20 | Lt | No | Solid | 11 | Serous | No | Yes | IIIC |
| 10 | 55/F | Post | No | 15x15 | Lt | Yes | Solid | 5.5 | Benign | - | No | I |
| 11 | 45/F | Post | No | 8x8 | B/L | No | M | 0.7 | Serous |  | Yes | IIIC |
| 12 | 35/F | Post | Yes | 7x7 | B/L |  | Solid | 11.25 | Serous |  | Yes | IIIC |
| 13 | 20/F | Pre | No | 10x10 cm | B/L | Yes | Cysic & Solid | 482 | Serous |  | Yes | IV |
| 14 | 50/F | Post | Yes | 15x10 cm | Rt. | Partially | Cystic & Solid | N | Serous |  | Yes | II |
| 15 | 45/F | Post | Yes | 12x15 | B/L | Yes | Cystic | 129 | Serous |  | Yes | IIIC |
| 16 | 46/F | Post | No | 5x5cm | Rt. | Yes | Cystic | 17.26 | Serous | No | Yes | II |
| 17 | 40/F | Post | Yes | 10x6 | Rt. | Yes | Cystic | 103 | Serous | No | Yes | II |
| 18 | 50/F | Post | Yes | 17x15 cm | Rt. | Yes | Variegated | 8.71 | Serous | No | Yes | III |
| 19 | 53/F | Post | No | 3.0x3.0 | B/L | Yes | Variegated | 532.3 | Serous | No | Yes | III |
| 20 | 45/F | Post | No | 7x5cm | Lt. | No | Solid Cystic | 41.8 | Serous | No | Yes | IV |
| 21 | 53/F | Post | No | 14x9x13cm | Rt. | Yes | cystic | 438 | Serous |  | Yes | II |
| 22 | 48/F | Post | No | 3x3 cm | Lt. | Yes | Solid | 563 | Serous | - | Yes | IV |
| 23 | 48/F | Pre | Yes | 10cm | B/L | Yes | Soild |  | Serous | No | Yes | IIIC |
| 24 | 35/F | Post |  |  | B/L | Yes | Solid-Cystic | 45 | Serous |  | Yes | IIIC |
| 25 | 65/F | Post | Yes | 7 x6 | Rt | No | Soild | 17.3 | Serous |  | Yes | IIIC |
| 26 | 40/F | Post | Yes | 9x8 |  | No | No mass | 45 | Serous |  | Yes | IIIC |
| 27 | 45/F | Post | Yes | 7x5cm | Rt | Mobile | Solid | 8 | Serous |  | Yes | III |
| 28 | 42\F | Post | Yes | Rt.20x20 Lt.15x15 | Both | Yes | Solid-Cystic | 56 | Benign |  | Yes | I |
| 29 | 38/F | Pre | Yes | RT 6X5, LT 6X7 | Rt | Restricted | Solid-Cystic | 517.1 | Serous | No | Yes | IIIC |
| 30 | 47/F | Post | No | Not palpable |  | No | Solid | 393 | Serous Adeno Ca | No | Yes | III |
| 31 | 42/F | Pre | No | Not palpable | B/L | Nil | Cystic | 21.55 | Serous Adeno Ca | No | Yes | III |
| 32 | 28/F | Pre | No | Not palpable | B/L | Yes | Solid-Cystic | 490 | Serous |  | Yes | IIIC |
| 33 | 60/F | Post | Nil | Nil | B/L | Nil | Cystic | 7.8 | High grade Serous Adeno Ca | No | Yes | III |
| 34 | 22/F | Pre | Yes | 15x25 | Rt | Mobile | Solid | 980 | Mucinous |  | Yes | III |
| 35 | 55/F | Post | Yes | 34x40x42x31x35x16cm | R/L | Yes | Variegated | 31.6 | Serous Adeno Ca | No | yes | III |
| 36 | 45/F | Post | Yes | 10x6cm 5x4 cm | R/L | Yes | Solid | 307.9 | Serous Adeno Ca | No | Yes | III |
| 37 | 36/F | Pre | Yes | 5x6 4x5 | B/L | Yes | Solid | 41.25 | Serous Adeno Ca | - | Yes | III C |
| 38 | 35/F | Pre | Yes | 8x6 | L | Yes | Solid | 54346 | Serous Adeno Ca | - | Yes | III C |
| 39 | 45/F | Post | Yes | 17 cm | L | No | Variegated | 121 | Serous Adeno Ca | - | Yes | III |
| 40 | 60/F | Post | Yes | 6x4 | R | - | Variegated | 306 | Serous Adeno Ca | - | Yes | III |
| 41 | 60/F | Post | Yes | 5x4cm | Both | Mobile | Solid | 112.4 | Serous Adeno Ca | - | Yes | III |
| 42 | 55/F | Post | None | 19x19x12 | B/L | Mobile | Variegated | 63 | Serous Adeno Ca |  | Yes | III |
| 43 | 55/F | Post | - | 10x10cm 55x3.5 | Rt.Lt. | Mobile | multi | 98.5 | Mucinous |  | Yes | III |
| 44 | 60/F | Post | Yes | 8x8cm | B/L | Mobile | complex | 45.1 | Mucinous | No | Yes | IIIC |
| 45 | 28/F | Pre | Yes | 5x5cm | B/L | NotPalpale | complex | 40.1 | Serous | No | Yes | Iib |
| 46 | 25/F | Pre | Yes | Rt. 5x3, Lt. 4x3 | B/L | Yes | Variegated | 1279-479 | Serous Adeno Ca | No | Yes | IIIC |
| 47 | 57/F | Post | - | 30x20cm | L | Yes | Cystic | 59.79 | Serous | - | Yes | III |
| 48 | 48/F | Post | Yes | 15x12x10x12x10x11 | B/L | Mobile | Variegated | 51 | Serous (Poorly diff.) |  | Yes | IV |
| 49 | 47/F | Post | No | 5x4, 6x3 | B/L | No | solid | - | Serous | no | Yes | IIIC |
| 50 | 40/F | Post | No | - | both | No | Solid cystic | 66 | Serous Adeno Ca |  | Yes | III |
| 51 | 50/F | Post | Yes | - |  | No | - | 51.26 | Serous |  | Yes | IV |
| 52 | 51/F | Post | No | 5x3,4x3 | B/L | No | Solid cystic | 42 | Serous |  | Yes | IV |
| 53 | 52/F | Post | No | 10x3 | B/L | No | Solid cystic | 90.3 | Serous |  | Yes | IV |
| 54 | 54/F | Post | No | 7.5x2.8 | single | No | solid | 88.55 | Serous |  | Yes | III |
| 55 | 37/F | Pre | No | 13.7x6 | B/L | palpable | complex | 353.6 | Papillary Serous |  | Yes | III |
| 56 | 45/F | Pre | No | 14x11x10 | pelvic | No | Solid cystic | 110.4 | Serous |  | Yes | III |
| 57 | 43/F | Pre | no | 12.5x14x12 | pelvic | No | Solid cystic | 105.9 | Papillary Serous |  | Yes | III |
| 58 | 35/F | Pre | no | 6x3,4x3 | B/L | No | Solid cystic | 56 | Serous |  | Yes | III |
| 59 | 55/F | Post | no | 10x8x9 | R | Fixed | variegated | 34 | Serous Adeno Ca |  | Yes | IV |
| 60 | 47/F | Pre | No | 8x6 | lt | No | Solid | 948 | Serous Adeno Ca |  | Yes | III |
| 61 | 40/F | Pre | No | 4x3 | B/L | mobile | variegated | 21.5 | Serous | No | Yes | III |
| 62 | 50/F | Pre | Yes | Rt 5x3, 10x10 | both | mobile | variegated | 196 | Serous | No | Yes | III |
| 63 | 42/F | Pre | No | small | lt | No | Solid | 44.2 | Papillary cyst | No | Yes | IA |
| 64 | 50/F | Post | No | 5x5cm | B/L | No | solid | 168 | Serous Adeno Ca | no | Yes | III |
| 65 | 50/F | Pre | No | 15cm | rt | Yes | variegated | 116 | Serous Adeno Ca |  | Yes | IIIC |
| 66 | 45/F | Pre | Yes | 12x15 | B/L | Yes | cystic | 129 | Serous |  | Yes | IIIC |
| 67 | 45/F | Pre | Yes | 10x6, 5x4 | r/l | Yes | solid | 207 | Serous Adeno Ca |  | Yes | III |
| 68 | 45/F | Pre | Yes | 4.7cm | B/L | Yes | variegated | 62.7 | Serous |  | Yes | III |
| 69 | 55/F | Pre | No | 3.5 | Lt | Yes | variegated | 25.3 | Serous Adeno Ca |  | Yes | III |
| 70 | 55/F | Pre | No | 12x12 | lt | Yes | solid | 6.0 | Benign | Yes | No | I |
| 71 | 38/F | Pre | No | 14x9x12 | Rt |  | Restricted cystic | 407 | Serous |  | Yes | II |
| 72 | 60/F | Post | Yes | 6x6 | Rt | Yes | cystic | 20.1 | Serous |  | Yes | II |
| 73 | 45/F | Pre | No | 10x4 | Rt | Yes | Solid cystic | 5.5 | Benign | Yes | No | I |
| 74 | 34/F | Pre | Yes | 14x9 | B/L | Yes | cystic | 53 | Serous (Poorly diff) |  | Yes | IV |
| 75 | 52/F | Pre | No | 6x6 | B/L | Yes | solid | 2.3 | Benign | Yes | No | I |
| 76 | 43/F | Pre | Yes | 5x8 | Rt | No | solid | 38 | serous | No | Yes | I |
| 77 | 37/F | Pre | No | 16x7x14 | B/L | Yes | solid | 205 | serous | No | Yes | I |
| 78 | 52/F | Post | Yes | 6x6 | Lt | Yes | solid | 115 | serous | No | Yes | III |
| 79 | 50/F | Post | Yes | 4x3 | B/L | Yes | variegated | 89.4 | serous | No | Yes | III |
| 80 | 40/F | Pre | No | 5x4 | B/L | No | Restricted cystic | 43.3 | serous | No | Yes | I |
| 81 | 56/F | Post | Yes | 8x6 | Rt | Yes | variegated | 60.2 | serous | No | Yes | IV |
| 82 | 35/F | Pre | No | 10x8x9 | Rt | No | Solid cystic | 280 | serous | No | Yes | I |
| 83 | 28/F | Pre | No | 4x3 | Lt | No | Solid cystic | 32.1 | Mucinous | No | Yes | I |
| 84 | 24/F | Pre | Yes | 6x6 | B/L | Yes | Solid | 22 | Mucinous | No | Yes | I |
| 85 | 29/F | Pre | No | 5x3 | Rt | Yes | Solid | 15.7 | Mucinous | No | Yes | I |

1. **Normal control samples**

| **Sample No.** | **Age** | **Menopausal Status** | **Description** | **Size** | **Histology** | **Sample ID** |
| --- | --- | --- | --- | --- | --- | --- |
| 1 | 47/F | Pre | Uterus with cervix with bilateral tube and ovary | 5x3x1 cm | Normal histology | 5503-08/15 |
| 2 | 52/F | Post | Uterus with cervix | 2x2x1 cm | Normal histology | 5603-07/15 |
| 3 | 56/F | Post | Bilateral tube and Ovary | 4x3x2 cm | Normal histology | 6025-39/15 |
| 4 | 49/F | Pre | Bilateral tube and Ovary | 2x1 cm | Normal histology | 20130331-5961 |
| 5 | 62/F | Post | Bilateral tube and Ovary | 6cm | Normal histology | 6041-43/15 |
| 6 | 64/F | Post | Uterus with cervix with bilateral tube and ovary | 10x7x4 cm | Normal histology | 5849-62/15 |
| 7 | 54/F | Pre | Uterus with cervix with bilateral tube and ovary | 2.5x1.5x0.5 cm | Normal histology | 6193-99/15 |
| 8 | 67/F | Post | Bilateral tube and ovary | 2x1x0.3 cm | Normal histology | 6420-27/15 |
| 9 | 65/F | Post | Uterus with bilateral tube and Ovary | 9x7x5 cm | Normal histology | 20130331-7026 |
| 10 | 63/F | Post | Uterus with cervix with bilateral tube and ovary | 3x1.5x0.5 cm | Normal histology | 6838-51/15 |
| 11 | 56/F | Post | Uterus with cervix | 4x2.5x1 cm | Normal histology | 7110-19/15 |
| 12 | 59/F | Post | Uterus with cervix with bilateral tube and ovary | 2.5x1x0.5 cm | Normal histology | 6891-95/15 |
| 13 | 61/F | Post | Uterus with cervix with bilateral tube and ovary | 4x3x1 cm | Normal histology | 2248-53/16 |
| 14 | 65/F | Post | Uterus with cervix | 2x1 cm | Normal histology | 2341-47/16 |
| 15 | 63/F | Post | Uterus with cervix with bilateral tube and ovary | 4x2x0.5 cm | Normal histology | 2573-77/16 |
| 16 | 48/F | Pre | Enlarged entire uterus with cervix | 3x2x0.5 cm | Normal histology | 20130331-2582 |
| 17 | 61/F | Post | Bilateral tube and ovary | 2x2x1cm | Normal histology | 6295-6303/15 |
| 18 | 58/F | Post | Uterus with cervix | 8x3x2cm | Normal histology | 21030331-1539 |
| 19 | 48/F | Pre | Uterus with cervix with bilateral tube and ovary | 2x1cm | Normal histology | 2773/16 |
| 20 | 62/F | Post | Uterus with cervix with bilateral tube and ovary | 3x2.5x0.5cm | Normal histology | 2896-2908/16 |
| 21 | 43/F | Pre | Uterus with cervix with bilateral tube and ovary | 2.5x2.5x0.5cm | Normal histology | 2935-40/16 |
| 22 | 50/F | Post | Uterus with cervix with bilateral tube and ovary | 12x12 | Normal histology | KGMU |
| 23 | 62/F | Post | Uterus with cervix with bilateral tube and ovary | 15x15 | Normal histology | KGMU |
| 24 | 60/F | Post | Uterus with cervix with bilateral tube and ovary | 20x20 | Normal histology | KGMU |
| 25 | 65/F | Post | Uterus with cervix with bilateral tube and ovary | 15x15 | Normal histology | KGMU |
| 26 | 30/F | Pre | Uterus with cervix with bilateral tube and ovary | 12x12 | Normal histology | KGMU |
| 27 | 72/F | Post | Bilateral tube and ovary |  | Normal histology | KGMU |
| 28 | 56/F | Post | Bilateral tube and ovary |  | Normal histology | KGMU |
| 29 | 64/F | Post | Uterus with cervix with bilateral tube and ovary |  | Normal histology | KGMU |
| 30 | 63/F | Post | Uterus with cervix with bilateral tube and ovary |  | Normal histology | KGMU |
| 31 | 54/F | Post | Bilateral tube and ovary |  | Normal histology | KGMU |
| 32 | 57/F | Post | Uterus with cervix with bilateral tube and ovary |  | Normal histology | KGMU |
| 33 | 60/F | Post | Bilateral tube and ovary |  | Normal histology | KGMU |
| 34 | 51/F | Post | Uterus with cervix with bilateral tube and ovary |  | Normal histology | KGMU |
| 35 | 55/F | Post | Uterus with cervix with bilateral tube and ovary |  | Normal histology | KGMU |

**Table S4: Performance of candidate marker panels in the prediction of ovarian cancer using serum samples.**

| **Marker panel** | **AUC** | **SE** | **95% CI** | **Performance comparison (DeLong’s Test)** |
| --- | --- | --- | --- | --- |
| **HIC+SOX1** | 0.928 | 0.0285 | 0.872 to 0.984 | Z= 2.370  p-value =0.0178 |
| **HIC+SOX1+CA125** | 0.975 | 0.0170 | 0.942 to 1.000 |  |
| **HOXA9+HIC1** | 0.948 | 0.0307 | 0.868 to 0.989 | Z= 2.056  p-value =0.0397 |
| **HOXA9+HIC1+CA125** | 0.977 | 0.0169 | 0.944 to 1.000 |  |
| **SOX1+HOXA9** | 0.857 | 0.0429 | 0.773 to 0.941 | Z= 3.025  p-value =0.0025 |
| **SOX1+HOXA9+CA125** | 0.974 | 0.0170 | 0.940 to 1.000 |  |
